# UAV and Deep Learning for Building Façade Defect Detection: A Comprehensive Review

**DOI:** 10.3390/s26123959

**Published:** 2026-06-22

**Authors:** Yue Fan, Yuheng Deng, Fei Xue, Jinghua Mai, Stephen Siu Yu Lau, Chi Ho Li

**Affiliations:** 1 State Key Laboratory of Subtropical Building and Urban Science, Center for Human-Oriented Environment and Sustainable Design, School of Architecture and Urban Planning, Shenzhen University, Shenzhen 518060, China; yfan@szu.edu.cn (Y.F.); 2510115054@mails.szu.edu.cn (Y.D.); 2310325038@email.szu.edu.cn (J.M.); ssylau@hku.hk (S.S.Y.L.); 2 Faculty of Architecture, The University of Hong Kong, Hong Kong 999077, China; 3 Department of Design and Architecture, Technological and Higher Education Institute of Hong Kong, Hong Kong 999077, China; chli@ieee.org

**Keywords:** unmanned aerial vehicle (UAV), deep learning, defect detection, path planning, multi-modal data fusion, defect identification, digital twin

## Abstract

Unmanned aerial vehicles (UAVs) and deep learning (DL) have introduced a new framework for intelligent building façade defect detection, yet existing studies often focus on isolated technical components and lack a systematic evaluation of the entire pipeline. To address this gap, this paper conducts a systematic literature review of 135 peer-reviewed journal articles retrieved from the Web of Science database over the period 2021–2026. This review investigates four key domains: (1) UAV inspection path planning and data acquisition; (2) multi-modal data fusion; (3) DL-driven defect detection algorithms; and (4) 3D reconstruction and digital twin integration. Our analysis reveals the following main findings. Real-time perception-aware planning is central to UAV path planning, yet most studies lack robustness evaluations under real-world deployment conditions. Multi-modal data fusion improves detection across multiple defect types, yet edge deployment requires balancing lightweight design with recognition stability. Defect recognition algorithms increasingly adopt task-driven architectures, but limited edge-device resources demand joint optimization of efficiency and accuracy. In digital twins, systematic research is still lacking on semantically integrating recognition results into BIM for O&M decision-making, leaving the closed loop from defect detection to maintenance unresolved. This review aims to help researchers and practitioners advance UAV-based inspection from an auxiliary tool to a fully autonomous, reliable intelligent agent for refined management of the urban built environment.

## 1. Introduction

Rapid global urbanization has made aging and deteriorating building façades a critical threat to public safety [1]. As key protective and functional components, façades are constantly exposed to complex environmental and anthropogenic stresses, including thermal cycling, acid rain, and mechanical loads, leading to defects such as cracks, spalling, and water seepage [2]. If undetected and unrepaired, these anomalies can cause cladding detachment and falling debris, resulting in severe casualties and substantial socioeconomic losses [3]. In dense high-rise areas, fallen façade tiles or masonry have become an urban high-altitude hazard, underscoring the urgent need for regular, efficient inspections [4]. Thus, timely and systematic façade inspection is essential to enable follow-up maintenance and reduce the risk of unexpected accidents.

Traditional building façade inspections rely on manual methods such as suspended gondolas, scaffolding, or rope access, which pose high operational risks, substantial costs, and limited viewing angles, often resulting in blind spots. These shortcomings hinder their ability to meet the demands of inspecting modern, ultra-large, and geometrically complex façades [5]. Recently, unmanned aerial vehicles (UAVs) equipped with advanced sensors have emerged as a breakthrough tool for digital façade inspection, offering superior maneuverability, multi-perspective data acquisition, and cost-effectiveness [6,7,8]. Concurrently, deep learning (DL)-based computer vision has achieved significant advances in image classification, object detection, and semantic segmentation [9,10,11,12,13]. Integrating state-of-the-art models like YOLO [14,15] and Mask R-CNN [16] with UAV platforms enables automated processing of massive inspection datasets and high-precision defect characterization, driving a fundamental shift from reactive response to proactive prevention [17].

Despite the great promise of integrating UAVs with DL, their practical deployment in complex construction environments still faces major hurdles. In urban canyons with dense high-rise buildings, GPS signal occlusion and complex geometric constraints severely limit autonomous inspection path planning [18,19,20]. Single-modal visible light sensors also fail to detect subsurface anomalies such as delamination or water seepage [21]; meanwhile, multi-modal data fusion (e.g., combining infrared thermography and LiDAR) still lacks generalization and real-time performance on edge devices [22,23,24,25]. Furthermore, a fundamental tension persists between the high computational demands of state-of-the-art DL models and the limited processing power of onboard UAV platforms [12,13,26]. Finally, most current defect detection outputs remain at the 2D image level and lack deep semantic linkage with Building Information Modeling (BIM), preventing a comprehensive closed loop from initial detection to maintenance decision-making [27,28].

Despite multiple reviews on UAV-based infrastructure inspection, three systemic gaps persist. First, existing surveys focus on isolated techniques, e.g., CNN-based defect recognition [13,29], computer vision optimization [30], or infrared thermography [21], without synthesizing the full chain of flight planning, multi-modal perception, intelligent recognition, and digital twinning. Second, most reviews target large-scale infrastructure (bridges, pavements, and power lines) but lack depth in building façades [6,7,20,31,32]. Third, critical practical challenges, such as real-time edge computing, onboard model lightweighting, and semantic digital twin integration, remain unaddressed [13,21,25]. While Cha et al. [12], Plevris and Papazafeiropoulos [29], and Lyu et al. [7] have advanced DL and AI in SHM, their broad scope dilutes domain-specific focus. Their focus fails to integrate UAV perception, edge inference, and semantic digital twins into a closed-loop architecture. Although some methods cited originate from other infrastructure, their underlying solutions are transferable. Thus, we anchor them to building façade defect inspection through four stages—path planning, multi-modal perception, defect recognition, and semantic digital twinning—forming an end-to-end framework.

**Figure 1:**
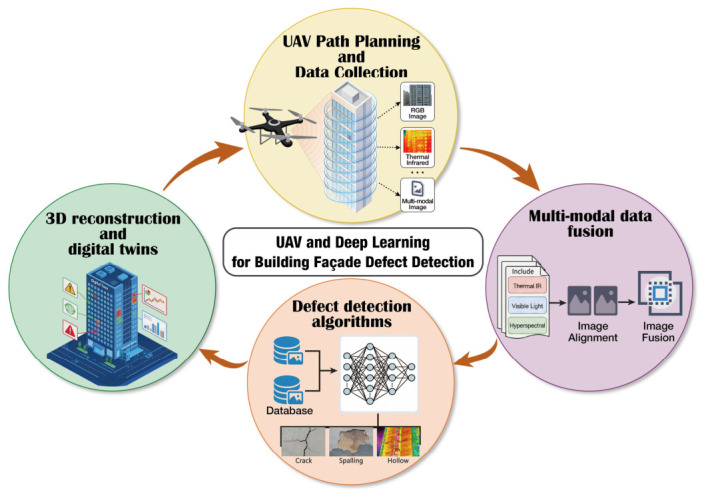
Technical flowchart of building façade defect detection based on UAV and DL.

Therefore, this review aims to bridge the research gaps by synthesizing the synergistic applications of UAV and DL in the field of building façade inspection. As shown in Figure 1, the core contributions of this study are delineated across four key dimensions:

- Path Planning Optimization: Analyzing the technological evolution of flight path planning from geometric optimization toward semantic-driven approaches;
- Multi-Modal Data Fusion: Exploring cross-sensor synergistic perception and its generalization strategies within complex environments;
- Accuracy–Efficiency Synergistic Recognition: Summarizing lightweight recognition algorithmic frameworks tailored for edge deployment;
- Full-Lifecycle Digital Twin: Elucidating the transformation of operation and maintenance (O&M) architectures from geometric reconstruction to semantic closed-loops.

The remainder of this paper is organized as follows: Section 2 introduces the methodology for literature retrieval and screening. Section 3 systematically reviews the research progress across the four key technical domains. Section 4 provides an in-depth discussion of current technical challenges and outlines future research trajectories. Finally, Section 5 concludes this review.

## 2. Methodology

This review is structured in accordance with the Preferred Reporting Items for Systematic Reviews and Meta-Analyses (PRISMA) 2020 guidelines. It aims to efficiently, comprehensively, and systematically synthesize research on UAV-based building defect inspection conducted over the past five years, thereby providing a foundational reference and strategic recommendations for future investigations. PRISMA is an evidence-based minimum set of items designed to enhance the reporting quality of systematic reviews, offering authors comprehensive guidance and illustrative examples on reporting the rationale, methodological frameworks, and derived outcomes of a review. Integrating the PRISMA flow diagram with the specific objectives of this study, this review was executed through a four-phase methodological framework: formulating research questions, literature collection, literature screening, and in-depth analysis.

### 2.1. Research Questions

To ensure the systematicity and comprehensiveness of this review, and to fully encompass the technical pipeline of UAV-based building inspection, specific research questions were formulated at the inception of this study. These questions are strictly aligned with the end-to-end logical workflow of “acquisition—perception—fusion—application”, guiding the entire literature retrieval and screening process while focusing on core technologies and critical challenges. The primary research questions include the following:

Q1: How can autonomous UAV flight inspection paths be optimally planned in complex, high-density urban environments to achieve efficient and precise acquisition of massive datasets?

Q2: How can multi-source heterogeneous data (e.g., visible light, infrared thermography, and LiDAR) be deeply fused to enable precise spatial localization and comprehensive diagnosis of defects?

Q3: Which state-of-the-art deep learning algorithms identified in the existing literature are most suitable for the intelligent recognition, diagnosis, and quantitative analysis of building façade anomalies (e.g., cracks, spalling, and water seepage)?

Q4: How can UAV-acquired defect recognition outputs be dynamically integrated into BIM to construct digital twin platforms that support full-lifecycle O&M?

Guided by these research questions, an in-depth investigation into the current state-of-the-art of UAV-based building inspection technologies was conducted, the findings of which are elaborated upon in the subsequent sections.

### 2.2. Literature Collection

**Figure 2:**
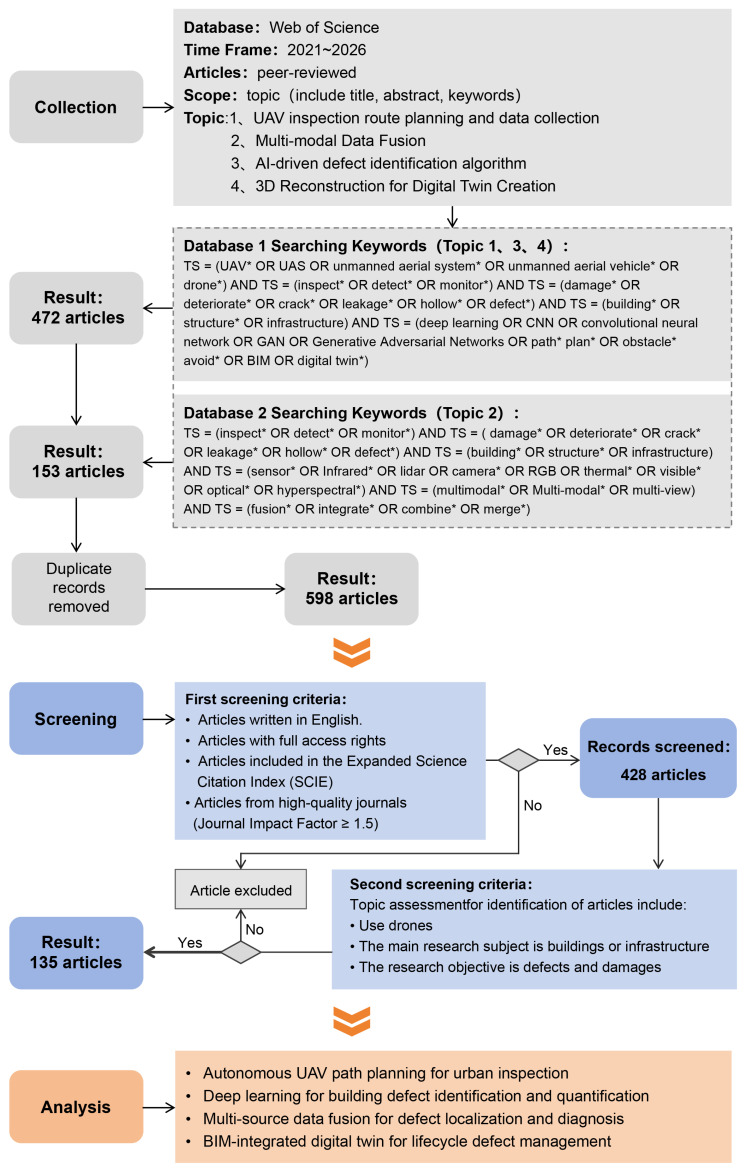
Flow chart of the research methodology.

This section meticulously details the retrieval protocol employed in this review for studies on UAV-based building inspection published over the past five years (2021–2026) (Figure 2). The specific parameters are defined as follows:

- Database: Web of Science (WoS) was selected as the primary search engine due to its extensive indexing of high-impact, peer-reviewed academic literature.
- Scope: To ensure the highest quality of sources, the collection was strictly limited to peer-reviewed journal articles and proceedings from top-tier international conferences.
- Timeframe: 2021–2026. The actual search date was 12 March 2026, and the specific search period covers the publication dates from 1 January 2021 to 31 December 2026. This chronological boundary was established to capture the most recent research and development trajectories of UAV technologies and deep learning algorithms in building defect monitoring, acknowledging their rapid and continuous iterative advancements.
- Keywords: The search strings formulated to delineate the scope of this review are detailed below:

Search String 1: UAV-based Intelligent Inspection and 3D Modeling
TS = (UAV* OR UAS OR unmanned aerial system* OR unmanned aerial vehicle* OR drone*)
AND TS = (inspect* OR detect* OR monitor*)
AND TS = (damage* OR deteriorate* OR crack* OR leakage* OR hollow* OR defect*) AND TS = (building* OR structure* OR infrastructure)
AND TS = (deep learning OR CNN OR convolutional neural network OR GAN OR Generative Adversarial Networks OR path* plan* OR obstacle* avoid* OR BIM OR Digital Twin*)

Search String 2: Multi-modal Sensor Defect Recognition and Data Fusion
TS = (inspect* OR detect* OR monitor*)
AND TS = (damage* OR deteriorate* OR crack* OR leakage* OR hollow* OR defect*)
AND TS = (building* OR structure* OR infrastructure)
AND TS = (sensor* OR Infrared* OR lidar OR camera* OR RGB OR thermal* OR visible* OR optical* OR hyperspectral*)
AND TS = (multimodal* OR Multi-modal* OR multi-view)
AND TS = (fusion* OR integrate* OR combine* OR merge*)

The keyword permutation combined core research themes with four predefined research questions. Keywords were classified as essential or directional. Essential keyword combinations formed four facets: platform, action, target, and object. Directional keywords were arranged by research direction. During retrieval, multi-source heterogeneous data fusion was not always tied to UAV themes in the literature but remained relevant to this review’s overall topic. Therefore, search String 2 was created separately to capture literature on multi-source heterogeneous data fusion. Boolean operators (AND, OR) were used to systematically combine keywords for targeted retrieval. This search strategy initially yielded 472 and 153 documents, respectively, retained for further screening.

### 2.3. Literature Screening

To ensure the high relevance of the retrieved literature to the research theme, a rigorous screening process was imperative. Initially, due to partial overlap between the results generated by the two search strings, the datasets were merged and deduplicated, yielding a consolidated corpus of 598 documents. This section delineates a two-stage screening protocol based on specific inclusion and exclusion criteria applied to the collected literature. The first stage of preliminary screening applied the following inclusion criteria:

- Studies published in the English language;
- Articles available in full-text access;
- Articles included in the Expanded Science Citation Index (SCIE);
- Articles from high-quality journals (Journal Impact Factor ≥ 1.5).

Following this initial screening phase, a total of 598 papers proceeded to the subsequent exclusion stage. The second stage involved an in-depth evaluation through the preliminary reading of abstracts and full texts to determine whether these papers satisfied the following eligibility criteria:

- Did the authors explicitly utilize UAVs?
- Were buildings or civil infrastructures the primary subjects of investigation?
- Was the primary research objective focused on structural defects or pathological anomalies?

Upon the completion of this rigorous two-stage screening process, a final corpus of 135 papers was selected for detailed review. The second-stage screening expanded its focus beyond building façades to include general civil infrastructure due to two main factors. Firstly, the limited existing research on UAV-based building façade inspection meant a comprehensive review would lack sufficient literature. Secondly, techniques and methodologies developed for other infrastructure, such as bridges and pavements, are transferable to building façade defect detection, offering valuable cross-disciplinary insights. While building façades remain the primary research focus, as shown in Table 1, other infrastructure types are also represented. Table 2 summarizes journals that contributed at least two papers to the final selection, reflecting their academic specialization and relevance to multidisciplinary researchers. *Sensors*, *Automation in Construction*, and *Journal of Building Engineering* were the most prominent publications in infrastructure inspection.

### 2.4. Literature Analysis

**Figure 3:**
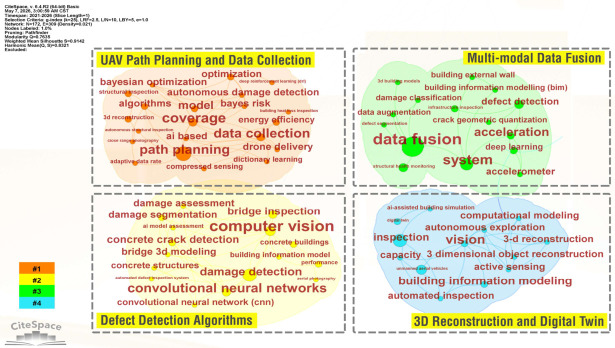
Keywords analysis and cluster mapping.

An in-depth, full-text analysis was conducted on the 135 included papers to extract critical information pertaining to UAV flight path planning, sensor data acquisition, multi-modal data fusion, defect recognition algorithms, and digital twin technologies. Overall, based on a thematic cluster analysis of the selected literature, four primary research categories were identified (Figure 3). These encompass the following:

- UAV flight path planning and data acquisition strategies within complex, high-density urban environments;
- DL-based algorithms for the intelligent recognition of building surface defects;
- Two-dimensional-to-three-dimensional fusion strategies for multi-modal data;
- BIM-integrated digital twin and the management of building O&M platforms.

The core research frameworks and technical specifics of each category will be comprehensively elaborated upon in the subsequent sections.

## 3. Results

### 3.1. UAV Inspection Path Planning and Data Acquisition

Section 3.1 explores three core approaches of UAV inspection path planning and data acquisition: offline planning, dynamic adaptive planning, and knowledge-driven planning. These methodologies are characterized by critical technical features, including geometric viewpoint optimization, real-time perceptual feedback, and anti-interference cognition. The integrated application of these strategies significantly enhances data acquisition quality and the level of inspection autonomy for UAV systems in complex environments.

#### 3.1.1. Path Planning Strategies and Viewpoint Optimization

UAV inspection path planning optimizes trajectories for full coverage, efficient flight, and high-quality imaging. Research focuses on geometric path optimization for shortest routes and viewpoint quality optimization for continuous, high-resolution acquisition [33]. The central challenge is balancing flight efficiency and viewpoint quality while guaranteeing complete target coverage.

First, geometric path optimization focuses on balancing path length, smoothness, and coverage completeness. Addressing the conflict between efficiency and coverage, Zhao et al. [34] proposed a two-stage decoupled optimization mechanism. This approach utilizes a Genetic Algorithm (GA) to optimize viewpoint positions in 3D space to minimize path length, followed by a greedy algorithm to solve camera poses satisfying field-of-view (FoV) constraints. By establishing full coverage as a hard constraint, this strategy effectively prevents missed detections in peripheral or occluded areas caused by the pursuit of flight efficiency, ensuring the reliability of inspection results. Regarding trajectory smoothness and mission efficiency, Tong et al. [35] introduced the Multi-Layer Angle-Distance Traveling Salesman Problem (ML-ADTSP), which significantly reduces flight acceleration through viewpoint height stratification and spiral path planning. For high-rise building inspections, Mathur et al. [36] utilized elliptical equations to generate standardized helical trajectories, achieving continuous bottom-up scanning and avoiding time wastage associated with vertical reciprocating movements. Furthermore, Hu et al. [37] introduced the Degree of Damage (DOD) index as a mission weight, optimizing multi-UAV inspection sequences via the Team Orienteering Problem (TOP), thereby facilitating an approach shift from geometric path optimality toward inspection value maximization.

Second, viewpoint quality optimization aims to resolve the contradiction between image clarity and coverage completeness. Using BIM as input, Wang et al. [38] introduced Ground Sampling Distance (GSD) and overlap rates as key metrics to automatically calculate optimal viewpoint positions and poses for various surface types (e.g., planar, convex/concave, and edges). This effectively transforms imaging requirements into quantifiable geometric constraints. To improve deployment efficiency, Tan et al. [26] employed a grid discretization strategy to generate viewpoints at grid centers; however, this approach faces limitations regarding viewpoint density imbalance in curved regions. For multi-scale detection requirements, Tong et al. [35] proposed a hybrid generation method combining revolving viewpoint and gap-filling viewpoint, enabling a single framework to simultaneously satisfy close-range crack recognition and long-range photogrammetry. Additionally, Song et al. [27] introduced a closed-loop feedback approach, dynamically adjusting subsequent viewpoints by online evaluation of 3D reconstruction quality, marking a transition from static presets to dynamic quality-driven planning.

#### 3.1.2. Dynamic Adaptive Planning

Offline path planning struggles with real-world uncertainties like unexpected obstacles or imperfect reconstructions. UAVs need to dynamically adjust paths based on real-time perception, transitioning from preset trajectories to a perception-decision-action loop.

In terms of detection-driven path adjustment, Tan et al. [26] proposed a “global-local” adaptive inspection method. This system utilizes onboard edge computing for real-time damage recognition to trigger localized refined scans, realizing a “coarse-to-fine” inspection approach. This achieves deep coupling between path planning and real-time detection, although global boundaries still rely on BIM presets. Tse et al. [39] integrated onboard YOLOv4 with RGB-D (depth) cameras to achieve unified damage detection and centimeter-level spatial localization; however, its reliance on external motion capture systems limits practical outdoor deployment. Regarding obstacle-driven dynamic planning, Waqas et al. [40] combined a lightweight YOLOv3 model with clustering algorithms for real-time obstacle recognition and detour waypoint generation. While this method meets real-time flight requirements, its avoidance strategy is constrained by preset target categories. Furthermore, due to the lack of depth information support, it struggles with precise 3D avoidance and exhibits limited perception of slender objects.

Regarding reconstruction-quality-based closed-loop optimization, Song et al. [27] integrated online Multi-View Stereo (MVS) reconstruction with a quality assessment mechanism, dynamically infilling viewpoints via real-time feedback to achieve self-perfection of 3D models within a single flight. This method utilizes reconstruction precision as a feedback signal to guide path adjustments, significantly enhancing model completeness, though high depth-estimation overhead limits its real-time deployment on lightweight platforms. In multi-UAV cluster coordination, Cui et al. [18] proposed a Large Language Model (LLM)-driven multi-agent collaborative framework. By utilizing a task auction mechanism for autonomous sub-task allocation and dynamic re-planning, it effectively addresses task continuity under single-agent failure. Experiments demonstrate that this framework can exponentially improve inspection efficiency and enhance swarm robustness, though LLM onboard inference latency and communication load remain primary bottlenecks for its practical application.

#### 3.1.3. Knowledge-Driven Cognitive and Robust Planning

Dynamic adaptive planning, while enabling immediate responses based on real-time perception, typically optimizes only single missions locally. This section explores methodologies that leverage contextual information from past inspections to optimize long-cycle missions and develops robust strategies for extreme conditions like GPS-denied environments and electromagnetic interference. The goal is to evolve from reactive instant perception to proactive prior cognition and anti-interference planning.

In cognitive intelligence-driven path planning, Liu et al. [41] proposed a context-aware coverage path planning based on Semantic-PolygonGraph. By fusing static BIM semantics with real-time inspection data, the acquisition focus is shifted from blind geometric coverage to high-risk areas, significantly enhancing data effectiveness, albeit limited by BIM model completeness. At the risk-guided level, Zeng et al. [42] introduced Bayesian risk theory, correlating structural failure probabilities with path planning to achieve mechanics-driven adaptive sampling density. Hu et al. [37] utilized the DOD index as prior knowledge to optimize multi-UAV routes via TOP, ensuring priority inspection of high-risk regions. Furthermore, Cui et al. [18] leveraged LLMs to enhance swarm cognitive intelligence, achieving semantic understanding of inspection requirements and multi-UAV collaborative scheduling, providing logical support for task allocation in complex scenarios.

Robust planning is crucial for deploying inspection technology in challenging conditions like GPS-denied areas and signal occlusion. To address localization failure, Ali et al. [43] utilized ultrasonic beacon system (UBS) to achieve decimeter-level positioning in non-GPS environments, though the signals are susceptible to ambient noise and the deployment range is constrained. To counter strong electromagnetic interference, Waqas et al. [40] introduced ArUco markers for pose estimation, significantly improving flight stability in electromagnetically complex environments, though their application heavily depends on the pre-deployment of markers. In environment perception enhancement, researchers have utilized depth information from RGB-D cameras to improve execution capabilities. Mathur et al. [36] achieved dynamic obstacle avoidance through point cloud and trajectory optimization to ensure flight safety, while Tse et al. [39] combined depth data with pose estimation to achieve centimeter-level spatial localization of defects. Both studies expand the application value of depth information from the dimensions of avoidance and localization, yet they share challenges such as limited ranging distance, high computational overhead, or dependence on external positioning systems.

To systematically delineate the technological evolution discussed in Section 3.1.1, Section 3.1.2 and Section 3.1.3, Table 3 compares representative methods across six critical dimensions: required input model, GPS dependence, obstacle avoidance capability, suitability for high-rise façades, computational cost, and technical maturity. This comparison indicates that future UAV inspection path planning is trending toward edge-deployable and perception–planning coupling.

### 3.2. Multi-Modal Data Fusion

Section 3.2 focuses on three core dimensions of multi-modal data fusion: multi-modal data spatial alignment, core fusion network architectures, and performance validation. These approaches leverage cross-modal registration, multi-level feature fusion, and robustness evaluation to overcome single-sensor limitations. The result is improved segmentation accuracy, 3D measurement precision, and system generalization for defect detection in complex building environments.

#### 3.2.1. Spatial Alignment of Multi-Modal Data

Spatial alignment serves as the foundational phase of multi-modal fusion. To address non-linear radiometric differences between infrared and visible light, as well as the failure of traditional algorithms (e.g., SIFT, SURF) caused by low-texture surfaces and repetitive patterns on building façades, current research explores robust spatio-temporal consistency solutions across three areas: multi-source image registration, multi-view panoramic stitching, and 2D-to-3D cross-dimensional alignment.

In terms of multi-source image registration, the focus lies on overcoming radiometric disparities to extract stable features. Liu et al. [44] utilized phase congruency to extract structural features and achieved sub-pixel automatic registration via edge extrema points, though feature repeatability remains constrained in low-contrast scenarios. Lin et al. [45] proposed the radiation-invariant SRIF algorithm, which effectively suppresses cross-modal radiometric differences through local intensity binary transformation (LIFT), albeit with high computational complexity that limits real-time processing. Wang et al. [46] achieved automated coarse registration using principal orientation assignment combined with fast CPDA corner detection. To balance feature point density with matching reliability, Shahsavarani et al. [47] proposed a trade-off mechanism between feature point thresholds and Non-Maximum Suppression (NMS), reducing errors to 0.37 pixels, though the generalization of this criterion across different sensor combinations requires further validation.

For the spatial consistency requirements of panoramic stitching from adjacent images, Shahsavarani et al. [48] integrated self-supervised feature detection with Graph Neural Networks (GNNs) to achieve multi-modal image stitching in low-texture and repetitive pattern scenarios. Regarding 2D-to-3D cross-dimensional alignment, Elias et al. [23] proposed a registration method independent of hardware synchronization. This approach synthesizes images via Simultaneous Localization and Mapping (SLAM) point cloud rendering for feature matching with real images and utilizes Perspective-n-Point (PnP) optimization to accurately map thermal infrared attributes onto point clouds. Experiments demonstrated that this method achieves centimeter-level translation accuracy, supporting high-precision 3D thermal imaging; however, its heavy reliance on initial point cloud quality and high computational latency limit real-time applications.

#### 3.2.2. Core Methodologies for Multi-Modal Fusion

The essence of multi-modal data fusion lies in achieving complementary advantages across multi-source information. To address the challenges of significant feature distribution disparities and the difficulty of extracting complementary information, this section reviews the key technical paths for constructing efficient and robust fusion models across three dimensions: performance comparison of fusion strategies, evolution of feature-level fusion architectures, and intelligent feature selection and enhancement mechanisms.

**Figure 4:**
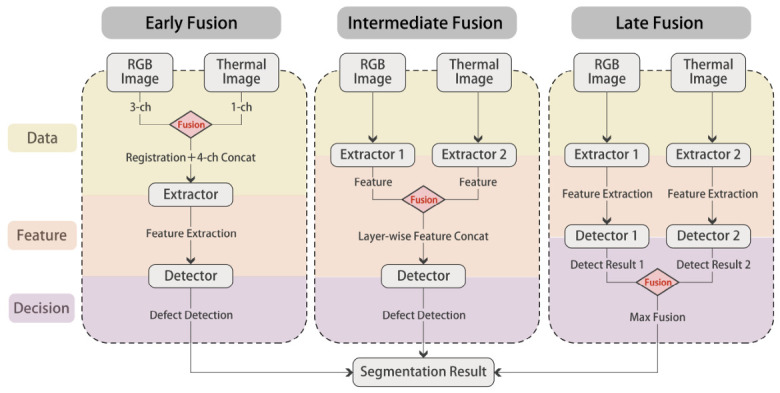
Three technologies for multi-modal data fusion.

In the performance comparison of fusion strategies, as shown in Figure 4, multi-modal data fusion techniques for RGB and thermal images can be divided into three levels: early fusion, late fusion, and intermediate fusion. Researchers have explored the suitability of different tasks based on the three stages. Comparative experiments by Yang et al. [49] indicated that early fusion (data-level) is more suitable for multi-defect detection on façades as it preserves complete raw information, whereas middle fusion (feature-level) performs better in segmenting cracks with simple textures by facilitating the extraction of discriminative features. Adriano et al. [50] and Pozzer et al. [51], respectively, validated the compensatory value of cross-modal combinations during data loss and the feasibility of using fused images as a substitute for unimodal data while maintaining segmentation accuracy, providing an empirical baseline for strategy selection.

In the evolution of feature-level fusion network architectures, research emphasizes the interaction and decoupling of deep information. The LBF2 Net proposed by He et al. [52] decomposes features into consistent and complementary components, effectively balancing the recognition of surface structures and internal anomalies, though its robustness is limited during modal quality degradation. To optimize detail preservation, Yuan et al. [22] and Zhao et al. [53] designed a structure-aware progressive multi-modal fusion network (SPMFNet) and a redetection multi-modal fusion network (RMFNet) based on Gated Control Attention (GCA), respectively, achieving incremental integration from local edges to global semantics. Furthermore, Hussain et al. [54] and Wang et al. [55] expanded the potential of fusion technology in diagnosing complex pathologies like moisture assessment through CSA Net dual cross-attention and multi-modal Convolutional Neural Network (CNN) framework.

The introduction of a Generative Adversarial Network (GAN) offers new perspectives for cross-modal feature alignment. Roheda et al. [56] utilized a Conditional Generative Adversarial Network (CGAN) to learn a cross-modal hidden space, ensuring system performance maintenance through spatial reconstruction even during sensor failure. Wang et al. [46] employed a dual-discriminator architecture to collaboratively constrain infrared thermal contrast and visible texture, significantly enhancing the visual fidelity of fused images, though training stability remains a challenge. Regarding intelligent feature selection and enhancement, research is pivoting toward adaptive evaluation and modality hallucination. Ren et al. [57] and Su et al. [58] introduced self-query Transformer and cross-modal guidance mechanisms to achieve dynamic feature weight adjustment based on content reliability, improving segmentation accuracy in low-light and noisy environments. The modality hallucination (MH) technique proposed by Mondal and Jahanshahi [59] supports the generation of depth features from RGB images alone, bypassing depth sensors during inference. Liu et al. [44] investigated PsFusion, which embeds semantic supervision to align the fusion process directly with downstream segmentation tasks, ensuring targeted enhancement of defect features in fused images.

Table 4 synthesizes the reviewed multi-modal fusion studies, comparing them across five dimensions: defect type, sensor modality, fusion architecture, advantages, and limitations. This clarifies the strengths, weaknesses, and applicability of various technical approaches.

#### 3.2.3. Performance Verification of Fusion Models

Performance verification serves as the quantitative benchmark for measuring the efficacy of multi-modal fusion models. Addressing the stability of complementary gains and generalization bottlenecks in complex scenarios, existing research primarily focuses on four dimensions: pixel-level segmentation accuracy, geometric measurement reliability, cross-domain generalization robustness, and downstream task performance.

In pixel-level segmentation accuracy, multi-modal fusion significantly improves the recognition rate of complex defects. Studies by Yang et al. [49] and Yu et al. [60] showed that the precision (97.44%) and mIoU (93.00%) of fusion models in façade and pavement detection far exceed unimodal performance. For thermal defect recognition, Chen et al. [61] reduced the false detection rate from 32% to 8.7% through fusion schemes; in the RGB-D fusion domain, Ren et al. [57] and Hussain et al. [54] both verified a stable mean Intersection over Union (mIoU) gain of approximately 4–5% from depth information. Although Pozzer et al. [51] ANOVA suggested that performance differences between modalities under specific CNN architectures might not be statistically significant, there is a consensus that the true value of fusion lies in the simultaneous detection of multiple defect types. However, high-precision results are currently concentrated on specific pathologies, and the generalization of models in scenarios with coexisting defect types remains to be tested.

At the geometric parameter measurement level, multi-modal fusion supports micron-level damage quantification. Ren et al. [57] and Hussain et al. [54] utilized RGB-D fusion to keep crack width measurement errors within 0.05 mm, with a relative error rate of only 7.3%. Lin et al. [45] compared three thermal texture mapping strategies and found that methods involving image pose optimization yielded the minimum root mean square error (RMSE) in 3D registration. In 3D spatial alignment, Shahsavarani et al. [47] achieved a registration accuracy of 0.37 pixels using Euclidean distance; Elias et al. [23] achieved centimeter-level translation standard deviation after registering images to SLAM point clouds, validating the high reliability of heterogeneous data in 3D representation. Nevertheless, current quantitative assessments rely heavily on ideal laboratory environments and lack large-scale validation under real-world engineering disturbances.

Regarding generalization and robustness in complex environments, the research focus is on addressing sensor failure and non-standard data. Liu et al. [44] demonstrated that the PsFusion model maintains an mIoU of 64.29% across multi-source device testing, showing excellent cross-domain generalization. To counter sensor damage or data loss, Pozzer et al. [62] utilized Siamese Neural Networks (SNNs) for post-processing CNN segmentation results, reducing false positives from 1192 to 561. Roheda et al. [56] ensured system availability during sensor damage through common hidden space reconstruction, and Adriano et al. [50] proved the compensatory efficacy of combining optical and Synthetic Aperture Radar (SAR) imagery in extreme disaster environments. While these robustness strategies significantly reduce false alarm rates, their high computational demands limit real-time response at the edge, making lightweight collaboration an inevitable trend.

In 3D spatial applications and downstream task performance, fusion technology has achieved a transition from image recognition to spatial semantics. Chen et al. [61] and Lin et al. [45], respectively, constructed point-cloud-level temperature integration models and 3D texture mapping workflows, intuitively revealing the three-dimensional distribution of implicit defects such as thermal bridges and seepage. Furthermore, Wang et al. [46] and Zhao et al. [53] achieved 85.4% and 91.1% mAP (mean Average Precision) in instance segmentation and object tracking tasks, respectively. Although modality hallucination (MH) technology has significantly enhanced segmentation performance, the overall 3D fusion outcomes currently remain in the model-construction phase, with a lack of deep integration into BIM and the application chain of full-lifecycle O&M [59].

### 3.3. AI-Driven Building Pathology Recognition Algorithms

Section 3.3 explores the technical evolution of AI algorithms for building pathology recognition. Key research areas include high-quality dataset creation and benchmarking, multi-scale recognition architecture optimization, lightweight edge deployment, and robust feature learning. These algorithms facilitate precise pixel-level segmentation of façade pathologies, automated quantification of geometric parameters (e.g., crack width and spalling area), and intelligent structural damage risk assessment (Table 5).

#### 3.3.1. Dataset Construction and Benchmarking for Algorithm Validation

Dataset construction and benchmarking are the fundamental cornerstones driving breakthroughs in DL for building pathology recognition [93]. Addressing challenges such as high labeling costs in real-world scenarios, the scarcity of long-tail samples, and inconsistent evaluation standards, existing research explores methodologies across three dimensions: engineering-oriented construction of real-world datasets, synthetic data with generative augmentation, and multi-dimensional standardized benchmarking frameworks.

In large-scale real-world dataset construction, the focus is on enhancing category diversity and annotation standardization. The MBDD2025 dataset released by Zha et al. [67] covers six structural categories and five defect types, ensuring high-consistency labeling through standardized zigzag flight path acquisition and multi-person cross-validation. Li et al. [112] constructed a concrete defect library exceeding 50,000 images and proposed an “information density” metric to quantify model parameter efficiency. While these works establish data scale as a core performance benchmark, they still face limitations such as center bias of defect targets and a lack of pixel-level fine annotation in massive datasets [15,67,112], which constrain the deep validation of semantic segmentation algorithms.

To bypass the bottlenecks of difficult data acquisition and high manual labeling costs, synthetic data generation based on digital twin and generative augmentation has become a vital supplement [105]. Yao et al. [109] utilized high-fidelity Computer Graphic (CG) models for automated pixel-level labeling and a “synthetic-driven, real-world fine-tuning” hybrid strategy to significantly reduce dependence on manual labels. Gwon et al. [98] and Wang et al. [66] employed Pix2PixHD image translation and StyleGAN v2 augmentation, respectively, to achieve a closed-loop for automated defect masks and resolve class imbalance, improving the F1-score for spalling recognition to 72.62%. Furthermore, Zhang et al. [117] extended a Deep Convolutional Generative Adversarial Network (DCGAN) generative augmentation to Ground Penetrating Radar (GPR) detection. However, the domain gap between synthetic and real-world data, alongside local distortions in GAN-generated samples, remains a primary obstacle to fully replacing real samples [66,96,109].

Regarding benchmarking frameworks and evaluation systems, research is shifting from single accuracy metrics toward a multi-dimensional trade-off of “accuracy–efficiency–robustness.” Zha et al. [67], Li et al. [112], and Meda et al. [72] provided engineering selection matrices covering CNN and Transformer architectures through unified evaluations of dozens of mainstream models, revealing that data quality often contributes more to performance than generational architectural iterations. Lightweight studies, such as the LCSNet proposed by Zhang and Huang [111], have demonstrated the necessity of incorporating model size, inference latency, and energy consumption into benchmarking systems. Despite significant progress, current benchmarks still face challenges such as insufficient cross-domain generalization assessment, lack of engineering metrics for edge devices, and unstandardized evaluation protocols for non-visible modalities like infrared and GPR [89,113,117,118].

#### 3.3.2. Evolution of Core Recognition Approaches and Architectural Optimization

The evolution of algorithmic architectures is the primary path to improving the quality of building pathology recognition. Addressing the progressive demands from qualitative classification to pixel-level segmentation, from apparent perception to implicit defect detection, and from high-performance models to edge deployment, current research focuses on methodologies such as classification, object detection, semantic segmentation, and lightweight backbone networks.

In qualitative classification and coarse localization, early research focused on establishing image-level discriminative capabilities. Le et al. [104] verified the feasibility of replacing manual inspection with automated interpretation via deep CNN; however, classification approaches struggle to provide precise spatial information. To bridge this gap, Kung et al. [103] introduced Class Activation Mapping (CAM), respectively, achieving a transition from existence determination to bounding box localization and heat-map guidance.

In pixel-level semantic understanding and dimensional transition, the research focus has shifted from regional recognition to contour delineation. Chen et al. [94] proposed a two-stage cascade architecture for classification-segmentation, utilizing a front-end classifier to suppress non-defect noise (e.g., windows, pipes) and significantly enhancing segmentation precision. Jin et al. [100] compared U-Net, DeepLabV3+, and TransUNet, leveraging the global modeling capabilities of Transformers to overcome the limitations of pure CNN in segmenting long, continuous cracks. Furthermore, Loverdos and Sarhosis [86] achieved a leap from 2D perception to 3D understanding, constructing 3D semantic point clouds via multi-view virtual rendering and back-projection, providing high-precision geometric carriers for structural health monitoring in digital twin environments.

In module-level refinement and feature enhancement, attention mechanism and operator optimization are key. The CSDNet proposed by Bhattacharya et al. [92] maintains high accuracy while reducing parameters by 80% through parallel processing of kernelized convolution and spatial-channel attention. Tse et al. [88] utilized frequency-domain analysis in USSA-Net to achieve a Dice coefficient of 0.968 on DeepCrack500. Kang et al. [119] integrated the Pyramid Pooling Module (PPM) from PSPNet with ASPP in parallel to strengthen the perception of subtle cracks and complex disaster zones. Additionally, Sarhadi et al. [83] proposed the T-Max-Avg learnable hybrid pooling layer to balance feature extraction depth and inference speed.

For real-time requirements and multi-target monitoring, single-stage detectors and ensemble learning strategies are widely applied [5,75,80,107]. Wang et al. [120] achieved 92.51% accuracy for airborne real-time detection of damaged buildings by improving the receptive field of YOLOv5s. Yang et al. [116] reconstructed the c2f module of YOLOv8 into an attention-enhanced structure for subtle leakage detection, improving mAP to 0.754. Yogi et al. [74] combined CNN with YOLOv5 for rooftop crack detection, achieving a 95.8% F1-score. To address accuracy bottlenecks in multi-scale tasks, Lee et al. [106] and Cai et al. [85] utilized ensemble learning and a three-stage “detection, segmentation, quantization” closed-loop to significantly improve IoU performance and measurement precision.

Table 6 summarizes the performance comparison of various semantic segmentation models reported by Han et al. [64], He et al. [81], and Amirkhani et al. [69] on the common DeepCrack dataset, in terms of four metrics: mIoU, Dice/F1-score, Precision, and Recall. DeepCrack, a widely used dataset in the field, comprises 537 crack images at 544 × 384 pixels, offering a diverse and comprehensive basis for model training and evaluation. However, comparisons in Table 2 should be interpreted cautiously due to variations in image resolution normalization, training/testing splits, hardware platforms, and evaluation metric calculations across the studies reviewed. These experimental conditions are detailed in Table 6 for reference.

Regarding implicit defects (e.g., air pockets, leakage), architectures are expanding toward multi-modal fusion networks. Li et al. [113] utilized a cascaded CS-YOLOv7 for debonding detection and a lightweight DeepLabv3+ for boundary segmentation, achieving an mAP of 89.5%. Wang et al. [46] designed a parallel fusion pipeline where infrared and visible images are fused via a dual-discriminator GAN after feature point registration, achieving an 85.4% mAP across four defect types. Fan et al. [17] constructed a collaborative visible–infrared detection framework for integrated 3D visualization of cracks and leakage. While each fusion architecture approach has its focus, challenges remain, including registration accuracy between heterogeneous sensors and diversity in fusion strategies [17,46,87,113].

The generational shift of backbone networks drives continuous evolution. He et al. [81] introduced the Mamba State Space Model, achieving global dependency modeling with linear complexity. Lethanh et al. [76] verified that Vision Transformers (ViTs) (98% accuracy) slightly outperform CNNs (95%). As performance reaches saturation, Bae et al. [63] and Han et al. [64] have turned to lightweight designs, achieving real-time inference on consumer-grade UAV while maintaining high F1-scores, facilitating the migration of algorithms from validation to engineering practice.

#### 3.3.3. Robust Feature Learning in Complex Scenarios

Robust feature learning is essential for enhancing the engineering reliability of defect recognition models. Addressing severe challenges such as complex background interference, imaging blur, limited resolution, and the scarcity of long-tail samples, this section analyzes technical paths across four dimensions, including architecture-level noise suppression, signal-level image restoration, adaptive learning strategies, and cognitive attention guidance.

In architecture-level noise suppression and signal restoration, research focuses on eliminating misjudgments caused by environmental noise [14]. To counter “crack-like” structural interference on façades, the two-stage cascade framework by Chen et al. [94] improved segmentation accuracy from 65.87% to 97.40%. The CrackSight model by Amirkhani et al. [69] utilizes deep fusion of detection and segmentation branches, combined with adaptive dilated convolutions and a dual attention mechanism, to enhance feature saliency in distant, complex backgrounds.

To address imaging blur and scale variation, researchers utilize signal compensation and multi-scale fusion. Xiang et al. [108] introduced Super-Resolution Reconstruction (SRR) as a pre-processing module, utilizing networks like the Super-Resolution Feedback Network (SRFBN) to restore low-resolution images, which significantly improved the IoU of crack boundary recognition. Wang et al. [84] utilized a U-Net generator and PatchGAN discriminator to raise crack IoU from 59.62% to 74.60% on blurred test sets. For scale changes caused by varying safety distances, Bae et al. [63] designed a scale-adaptive optimization module using multi-scale pyramid weighted fusion. Additionally, Jiang et al. [99] introduced point cloud elevation and normal features to enhance the separability of cracks from backgrounds via 3D geometric priors.

Regarding single-pixel weak signals and sample scarcity, learning strategies are evolving toward high sensitivity and data efficiency. Geetha et al. [121] combined 1D (Discrete Fourier Transform) DFT-CNN with iterative differential sensitivity tracking for the effective recall of hair-line cracks. In few-shot learning, Zhong et al. [90] and Xu et al. [78] introduced meta-learning mechanisms to ensure rapid migration to new damage categories with minimal labeling. Meanwhile, Agyemang et al. [122] and Tauzowski et al. [77] utilized reinforcement learning with visual rewards and a fully synthetic dataset, respectively, to mitigate long-tail distribution issues.

Facing the challenge of defect sparsity in large-scale inspections, Tan and Li [114] proposed a cognitive perception framework that simulates professional scanning patterns through an attention-guided inspection path (AIP) generated by a perceptual fuzzy system. Meng and Wang [65] utilized a UE/UF feature selection framework to explicitly embed computational costs into optimization goals, doubling inference speeds on the 650,000-image LSBS dataset.

#### 3.3.4. Lightweight Model Design for Edge Deployment

Lightweight model design is the core driver for UAVs to transition from image acquisition terminals to autonomous real-time inspection platforms [101,123]. Addressing the bottlenecks of traditional models’ reliance on offline GPU inference and the constraints of embedded edge-side power, research explores three dimensions, including efficient backbone design, semantic segmentation architecture compression, and system-level algorithm-hardware synergy.

In early explorations of onboard real-time inference, researchers verified the feasibility of lightweight architectures on embedded platforms. Early high-precision models (e.g., DetectorX) required RTX 3090 GPUs due to computation loads reaching 89.2B FLOPs [122]. Kumar et al. [102] and Tse et al. [39] achieved initial real-time responses (0.19 s/frame) on the Jetson TX2 platform using YOLOv3-Tiny and YOLOv4-SE. These works established the structures of streamlined architecture and local attention.

In efficient backbone selection and model compression, researchers balance computational load through architecture pruning and transfer learning. Nguyen et al. [82] showed that MobileNetV2 exhibits superior edge compatibility in terms of size (3.2 MB) and latency (12 ms). Wang et al. [120] and Altaf et al. [68] further reduced model parameters to the 13.7 M range by removing redundant modules and introducing residual dilated convolutions or a weighted cross-entropy loss function, enabling deployment on ultra-low-power devices like the Raspberry Pi 4. In high-performance semantic segmentation compression, the focus has shifted to pixel-level perception with minimal parameters. LCSNet, proposed by Zhang and Huang [111], utilizes learned group convolutions (LConv) and shifted multi-direction convolution (SMDCM) to achieve a 90% FLOPs reduction with only 2 M parameters. Han et al. [64] proposed UMDA, which uses MobileNetV3 as an encoder to achieve a high frame rate of 61.2 FPS on a CPU, completing closed-loop validation on the DJI Mini 3. Table 7 compares lightweight models from six studies, evaluating their deployment suitability. While some models like MobileVNet offer advantages in size and latency, they lack real-world edge-device testing. Conversely, models like UMDA, CladdingNet, and Tiny YOLOv3 have been field-tested but exhibit low frame rates (<10 FPS) due to hardware or environmental limitations.

Regarding system-level synergy and perception-triggered optimization, researchers aim to reduce edge burdens through computational strategies. Egodawela et al. [97] proposed “coarse to fine” cascaded architectures and an ultrasonic sensor triggering mechanism to avoid ineffective computation. Gonthina et al. [70] integrated improved YOLOv8 segmentation with crack quantification and a six-level severity grading system on a Raspberry Pi 4 for one-stop edge assessment. Huang et al. [75] deployed CladdingNet on a Jetson AGX Xavier, where, by integrating 3D path planning with binocular stereo ranging, onboard real-time segmentation of cracks in stone cladding was achieved. In contrast, the “edge acquisition + cloud analysis” mode adopted by Silva et al. [15] utilizing YOLOv12 remains dependent on network connectivity, limiting its use in a communication-denied environment.

#### 3.3.5. Defect Geometric Parameter Estimation and Grading Mechanisms

Geometric parameter estimation and grading are critical for transitioning structural health monitoring from pathology perception to O&M decision-making. Research aims to overcome projection distortion, scale variation, and inconsistent standards to achieve accurate geometric acquisition and grading.

In 2D image quantification, the focus is on robust pixel-to-physical scale transformation models. Cai et al. [85] and Zhang et al. [80] achieved crack width measurement precision better than 0.15 mm using morphological skeletonization and Hough transform modeling combined with pinhole camera calibration. Gonthina et al. [70] implemented lightweight size estimation and a six-level dual-dimension grading system (length and width). Meda et al. [72] proposed using distance transforms for crack width estimation and area ratios for spalling and corrosion. Furthermore, Li et al. [113] and Yao et al. [124], respectively, utilized multi-modal fusion and color space transformation techniques to extend the quantification targets from surface cracks to thermal defect areas and concrete chromatic aberration. While 2D methods are efficient, their reliance on the “optical axis perpendicular to the wall” assumption makes them prone to perspective distortion during UAV maneuvers.

In 3D spatial quantification and localization, point cloud reconstruction breaks the accuracy limits of planar projection. Huang et al. [71] utilized spherical region search and Poisson reconstruction algorithm to achieve automated measurement of crack widths (0.09 mm average error) and spalling volumes. Tse et al. [39] and Choi et al. [95] utilized RGB-D perception and LiDAR-SLAM for centimeter-level real-time localization and geometric characterization. To support digital O&M, Yang et al. [79] and Gan et al. [16] achieved precise correlation of defects from component-level digital twin to city-level georeferencing through Real-Time Kinematic (RTK) GPS and BIM mapping. Furthermore, Yoon et al. [110] proposed a lightweight 3D modeling method based on geographic information system (GIS) corner point coordinates, which is suitable for rapid surveys of large-scale building clusters. Santos et al. [115] generated structural façade maps with rebar exposure markers via AlexNet sliding window detection and orthophoto mosaic, providing an intuitive tool for assessing the spatial extent of severe defects.

In grading mechanism construction, the trend is toward establishing standardized threshold models. Wang et al. [66] improved the F1-score for a five-level spalling classification to 72.62% using DamageNet. Woo et al. [33] distinguished between structural and non-structural cracks by overlaying YOLOv5 results with Computer-Aided Design (CAD) layers. The quantification threshold models proposed by Cai et al. [85] and Gan et al. [16] are particularly significant for engineering, as they map measured parameters directly to industry codes (e.g., CECS 293 or JTG bridge assessment standards) to output authoritative maintenance decisions. However, technical bottlenecks remain in multi-defect coupled assessment and cross-specification adaptability.

### 3.4. 3D Reconstruction and Digital Twin

Section 3.4 details the integrated use of 3D reconstruction and digital twin (DT) technologies for building defect management. These technologies encompass geometric reconstruction via photogrammetry and learning-based MVS, precise registration utilizing coordinate transformation and ray casting, and semantic mapping based on ontological modeling and multi-source data integration. The methods enable accurate migration and parametric representation of building defects from 2D images to 3D digital twin, establishing a digital platform for closed-loop structural health monitoring O&M.

#### 3.4.1. 3D Reconstruction as a Defect Mapping Carrier

Three-dimensional geometric models are fundamental for precise defect mapping. Current research focuses on three approaches to overcome challenges like occlusion in complex geometries, high computational costs in large scenes, and the sensitivity of Structure from Motion (SfM) to low-texture surfaces: photogrammetry-based mesh reconstruction, multi-source laser scanning, and learning-based MVS with real-time reconstruction.

In photogrammetry-based mesh reconstruction, the SfM-MVS algorithm remains the mainstream choice for constructing mapping carriers. Yigit and Uysal [125] and Jiang et al. [126] utilized UAV-derived multi-view imagery to generate Level of Detail (LOD) three-level refined models and semantic point clouds, achieving high-precision component recognition. Although cost-effective and automated, this path often suffers from point cloud “holes” or geometric distortion in low-texture areas (e.g., glass curtain walls) [127]. To mitigate this, Levine et al. [128] and Levine and Spencer [129] introduced Physics-Based Graphics Models (PBGMs) to enhance modeling fidelity in synthetic environments, providing a reliable geometric benchmark for algorithm validation.

To overcome photogrammetry’s dependence on environmental texture, laser scanning and multi-source fusion offer superior robustness. Shen et al. [130] utilized point cloud data to compensate for the depth-perception deficiencies of infrared thermography on irregular surfaces, significantly improving anomaly detection. Polania et al. [131] and Yoon et al. [110], respectively, explored high-fidelity mesh-to-BIM restoration and rapid modeling based on GIS corner points—the former focusing on extreme geometric detail and the latter on time-sensitive emergency surveys. Despite their precision, high equipment costs and computational overhead limit their large-scale adoption.

DL and online reconstruction are redefining efficiency boundaries. Yang et al. [79] demonstrated that learning-based MVS outperforms traditional algorithms in reconstructing low-texture regions. Notably, the active path planning and online MVS framework proposed by Song et al. [27] implements a “reconstruct while plan” closed loop by evaluating model completeness in real-time, serving as a technical template for the transition from static modeling to dynamic perception in autonomous inspections.

#### 3.4.2. 3D Coordinate Transformation and Spatial Registration

Spatial mapping and geometric alignment are the core links for transforming 2D recognition results into 3D spatial semantics. Addressing projection deviations caused by camera pose jitters, coordinate system mismatches between multi-source data, and occlusion misalignments on complex façades, research focuses on geometric registration via coordinate transformation, visual localization via ray casting/depth maps, and BIM-semantic-aided alignment.

Geometric registration via coordinate transformation aims to bridge the gap between UAV sensor data and engineering coordinate systems. Hao et al. [132] constructed a transformation matrix from the World Geodetic System 1984 (WGS84) global coordinate system through Earth-Centered, Earth-Fixed (ECEF) and Northeast Sky (NEZ) to the Revit local coordinate system. For geometrically regular buildings, Tan et al. [133] and Yoon et al. [110] utilized the Haversine formula and azimuth angles to generate virtual points, correcting deviations caused by the Earth’s curvature. While these methods achieve millimeter-level localization in open-signal environments, they are highly susceptible to GPS instability in communication-denied areas (e.g., bridge undersides).

To bypass satellite navigation limitations, visual localization via ray casting and depth maps has been widely explored. Zhang et al. [134] and Mathur et al. [36] modeled virtual cameras using intrinsic/extrinsic parameters and utilized ray-to-model intersection techniques with distortion correction to achieve centimeter-level registration. Building on this, Tan et al. [26] designed a “Global-to-Local” adaptive framework, utilizing secondary zoom-camera triggers and depth-map calculations for progressive refinement from coarse localization to fine registration.

BIM-semantic-aided alignment strategies significantly improve accuracy for complex façades. Levine et al. [128] innovatively utilized unique semantic IDs of BIM components to generate color-coded masks, combined with GrabCut segmentation to iteratively correct projection errors, raising the defect localization IoU above 0.80. Furthermore, by calculating Euclidean distances between pre- and post-seismic point clouds, they achieved non-texture localization for large-scale damage like structural collapse. While BIM priors reduce cumulative error, the computational cost of high-frequency iterations remains a bottleneck for edge-side real-time applications [129].

#### 3.4.3. Semantic Mapping of Defect Information

Semantic mapping is the essential link for converting recognition results from visual features into structured knowledge. Addressing the lack of physical attributes in pixel-level masks and the logical gap between detection data and digital twin carriers, research focuses on 3D semantic association, standardized ontological modeling, and parametric representation.

Three-dimensional semantic association and annotation focus on the precise pairing of visual results with geometric entities. Huang et al. [71] and Yigit and Uysal [125] directly extracted defect geometries from 3D model surfaces or semantic point clouds, avoiding the geometric distortions inherent in traditional texture mapping. Yang et al. [135] proposed Surface Defect-Extended BIM (SDE-BIM) to explore pixel-level projection paths, completing the critical transformation from image masks to component attributes. Shen et al. [130] used point cloud fusion for semantic representation of thermal defects in non-visible light, establishing a foundation for defect analysis that moves beyond pixel-level labeling to incorporate spatial-geometric correlation.

Standardized classification and modeling are evolving from static data recording toward knowledge-based logical reasoning. To meet O&M standardization requirements, Nieto-Julián et al. [136] introduced national classification standards to establish mapping systems between pathologies and Historic Building Information Modeling (HBIM) components. To handle complex logic, Hamdan et al. [137] constructed a multi-layered ontology framework (OWL) and utilized Shapes Constraint Language (SHACL) rules for automated classification. This Knowledge Graph framework enhances model interpretability and supports intelligent decision-making in data-scarce scenarios.

Structured storage and parametric representation aim to build efficient, interactive digital O&M archives. Gan et al. [16] and Tan et al. [133] utilized Revit parametric families to solidify physical attributes (length, width, and depth) into editable geometric parameters. For large-scale storage, Han [138] and Matos et al. [28] proposed lightweight schemes based on Industry Foundation Class (IFC) Property Sets and placeholder family, significantly reducing computational overhead while maintaining information integrity. These solutions provide various technical approaches for managing defects throughout the lifecycle of buildings and infrastructure.

#### 3.4.4. Multi-Source Data Integration for Digital Twin

Multi-source data integration is the final step in driving building pathology into the management decision-making phase. Research explores high-interoperability solutions across three dimensions, including BIM-centric single-platform integration, international standard-based cross-model fusion, and lifecycle O&M platforms.

BIM serves as the unified data container in single-platform integration. Han [138] and Yang et al. [135] achieved lightweight presentation of defects within BIM via IFC property sets and surface texture mapping. To enhance automation, Tan et al. [133] and Gan et al. [16] utilized Dynamo scripts to automatically associate geometric parameters with component properties. Nieto-Julián et al. [136] and Polania et al. [131] investigated industry-standard semantic integration and high-fidelity mesh-to-BIM conversion in HBIM and existing buildings. While highly compatible with industry workflows, these paths face bottlenecks in model bloating when handling massive datasets [129].

International standard-based multi-model integration provides superior interoperability. Hamdan et al. [137] followed the ISO 21597-1 standard, utilizing Information Container for Linked Document Delivery (ICDD) to construct independent linked models. This loose coupling method keeps IFC models, point clouds, OWL damage ontologies, and Finite Element Models (FEMs) independently stored at the physical layer while establishing logical mappings at the semantic layer.

Lifecycle O&M platforms represent the ultimate form of integration, aiming to close the loop from detection to decision. Jang et al. [139] and Matos et al. [28] integrated 4D/5D management and dynamic damage grading within a BIM-FM environment. Zhang et al. [134] introduced a WebGIS platform that, through the cross-scale integration of BIM and a lightweight geographic information system, solves centimeter-level visualization challenges in city-scale contexts. These platforms provide the digital twin foundation necessary for Building Lifecycle Management (BLM).

## 4. Discussion

While the integration of UAV and DL has markedly advanced the capabilities of building façade pathology recognition, several unresolved technical bottlenecks continue to impede seamless deployment in practical engineering scenarios. Accordingly, this section provides an in-depth analysis of four core limitations constraining current technical translation, including insufficient autonomy in inspection trajectory planning; intelligence constraints within multi-modal data fusion; inadequate architectural synergy in recognition algorithms; and the absence of a technical closed-loop within digital twin-based O&M systems.

By delineating forward-looking research trajectories to address these hurdles, this section explores the necessary paradigm shift for UAV-based façade inspection—evolving from “auxiliary detection tools” focused on isolated optimizations toward “engineering-grade intelligent agent solutions” characterized by full-link coordination. Such an evolution is critical to providing a robust technical foundation for the safety management and intelligent O&M of the existing urban building stock.

### 4.1. Current Gaps and Challenges

While UAV-based façade pathology inspection has achieved phased progress across path planning, multi-modal fusion, recognition algorithms, and 3D reconstruction, significant challenges and research gaps persist. This section systematically analyzes the core difficulties encountered during the transition of the technical chain from experimental environments to engineering field applications across the four aforementioned technological perspectives.

#### 4.1.1. Limitations in UAV Inspection Path Planning

Despite progress in geometric path optimization, dynamic adaptive adjustment, and cognitive-robust planning, several constraints hinder practical engineering deployment [34,35]. A key limitation is the heavy reliance on precise prior models. Current methods assume static structures and require BIM/CAD data, but most legacy buildings lack digital documentation and feature modifications or temporary obstructions, making offline planning impractical [26,38,41]. Additionally, geometric path optimization and viewpoint optimization are often decoupled: the former prioritizes flight efficiency via path compression, while the latter increases viewpoint density for imaging quality. This trade-off lacks a unified theoretical framework, preventing globally optimal inspection performance [34,35,38].

Another limitation is insufficient robustness in perception and decision-making. Most dynamic adaptive planning uses a single sensing modality, which degrades under extreme illumination, adverse weather, or electromagnetic interference. Obstacle avoidance and re-planning are largely rule-driven, lacking adaptive capacity to scene complexity [39,40]. Closed-loop optimization based on online reconstruction quality feedback is conceptually advanced but requires complete MVS depth estimation, whose computational overhead conflicts with real-time constraints, hindering deployment on lightweight platforms [27]. For multi-UAV swarms, communication latency and bandwidth bottlenecks scale with the number of agents, limiting centralized scheduling architectures [18].

Furthermore, the integration of cognitive intelligence and robust planning remains superficial. Historical inspection data is used rudimentarily, and advanced technologies like LLMs show potential but suffer from onboard inference latency and communication overhead that do not meet real-time requirements [18]. Robust localization schemes also have boundary conditions: ultrasonic beacons have limited range, visual markers depend on lighting and distance, and depth cameras face a trade-off between range and computational cost. No single technology is universally applicable in GNSS-denied environments [39,40,43]. Thus, breakthroughs are needed in three areas: model-free operation, multi-modal fusion, and edge intelligence synergy.

#### 4.1.2. Limitations in Multi-Modal Data Fusion

Multi-modal data fusion has demonstrated significant potential in detecting various pathology types and can serve as a high-fidelity alternative to unimodal imagery. However, its practical application faces systemic challenges, primarily regarding the limited automation of data pre-processing and the poor generalizability of existing fusion strategies. The spatial alignment of multi-modal data during preprocessing is hampered by data quality, specifically due to sensor calibration, temporal synchronization, and thermal drift. Sensor calibration, typically performed in labs, suffers in the field from vibrations and temperature changes that cause parameter drift and sensor misalignment, resulting in thermal image distortion and inter-modal biases [45,61]. Temporal synchronization issues arise from differing sampling rates, where even millisecond-level asynchrony can lead to significant spatial misalignment, especially in high-speed applications, thus degrading fusion performance [23]. Thermal drift in uncooled thermal imagers, caused by ambient temperature changes or self-heating, undermines consistent thermal defect quantification [45]. While often overlooked in algorithm development, these issues are major obstacles to deploying multi-modal fusion systems in practice.

Secondly, existing fusion strategies lack unified theoretical guidance, resulting in increasingly complex network architectures with poor generalizability. Divergence exists between early-fusion and mid-fusion strategies, as each exhibits distinct advantages depending on the task scenario, often forcing researchers into extensive trial-and-error to determine the optimal approach [50,60]. Certain mid-level (feature-level) fusion networks, such as LBF2-Net and SPMFNet, suffer from high parameter counts and slow inference speeds, imposing a heavy computational burden on edge-side deployment [22,52]. Methods such as Generative Adversarial Networks (GANs) and modality hallucination improve robustness but suffer from training instability and hyperparameter sensitivity, limiting their widespread adoption. Most fusion methods are designed for specific sensor combinations, lacking a universal framework; consequently, the network must be redesigned whenever sensor types change, illustrating the lack of broad generalizability [49,56].

#### 4.1.3. Limitations in Building Pathology Recognition Algorithms

The reliability of recognition algorithms in real-world engineering scenarios is limited by poor generalization, high data dependency, and constrained edge deployment. Although models perform well in controlled settings, they suffer from significant performance drops in practice, lacking robustness against environmental disturbances such as sudden illumination changes and motion blur, with accuracy sharply declining under low-light conditions [81,94,104,120]. Multiple studies have quantitatively reported the impact of illumination variations on segmentation results. The LGFAF-Net proposed by He et al. [81] achieved a Dice coefficient of 93.7% under well-lit conditions, which dropped to 89.2% under dim lighting, primarily due to shadow effects and reduced crack visibility. Similarly, the UMDA model by Han et al. [64] exhibited performance degradation under adverse conditions such as strong backlighting or low illumination. These results consistently show that recognition accuracy declines when lighting conditions differ from those in the training data.

Existing algorithms are typically optimized for specific façade materials and defect types, leading to significant drops in detection precision when applied to different surface textures or novel damage categories [46,63,103]. This highlights a clear lack of cross-domain generalization beyond the training data distribution.

In terms of training approach and data dependency, current methods rely heavily on expensive and time-consuming pixel-level annotated datasets [67,112]. Although synthetic data and generative augmentation can reduce labeling costs, the domain gap between synthetic and real scenes remains a major barrier to replacing real-world data [66,96,109]. Additionally, most training and validation datasets assume fixed acquisition distances and ideal lighting, differing sharply from the viewpoint variations, distance fluctuations, and weather interference inherent in actual UAV inspections [100,120].

In terms of real-time performance versus accuracy, high-precision semantic segmentation models often exceed the computational capacity of embedded UAV devices. Lightweight architectures have advanced—achieving 2M parameters and over 60 FPS on CPU—but most studies only validate accuracy in GPU environments. Quantitative assessments of INT8 quantization, actual embedded latency, and system-level power consumption are generally missing [15,39,64,111]. This inherent trade-off between lightweight design and robustness continues to hinder the transition from laboratory prototypes to engineering practice [113].

#### 4.1.4. Limitations in 3D Reconstruction and Digital Twin Technologies

The ultimate goal of 3D reconstruction and digital twin for building defect mapping is to create a lifecycle digital twin O&M system. However, key bottlenecks persist across the technical chain. First, spatial registration of images in 3D space lacks robustness and automation; common coordinate transformation methods relying on GPS signals often fail in dense urban areas [132,133]. Ray casting and depth-map-based localization do not rely on GPS, but their accuracy is directly affected by depth-map quality and pose estimation errors [36,134]. BIM-aided registration can correct cumulative errors but requires high model completeness [129]. Errors accumulate across coordinate transformation stages, yet no systematic model quantifies their impact on final registration accuracy. These errors cause 3D localization biases in defect positions and inter-batch discrepancies. Current evaluations using mean error (ME) or root mean square error (RMSE) fail to characterize error distribution or link geometric misalignment to semantic annotation errors.

Following registration, automation and standardization of 3D semantic mapping for defect information need improvement. The automatic semantic association between DL-generated pixel-level masks and BIM components is lacking, so converting recognition results into manageable digital objects remains highly manual [71,125]. Poor interoperability across defect classification systems (e.g., SCFclass V2, ontological frameworks) hinders data fusion and reuse [136,137]. For batch storage of defect information, parametric families and property sets each have pros and cons, but no unified solution balances geometric visualization with lightweight management [16,133,138]. Moreover, most research relies on static single-inspection data, lacking support for temporal correlation and degradation trend analysis across multi-period detection data [28,137].

Regarding multi-source data integration for digital twin O&M, research is split between single-platform and multi-model integration, both with inherent limitations. BIM-centric single-platform integration tends to cause model bloating and reduce multi-disciplinary collaboration efficiency [16,133]. Although ICDD-based multi-model integration standardizes data association, its exchange efficiency and maintenance costs fail to meet real-time engineering needs, limiting scalability in large-scale O&M scenarios [137]. Full-lifecycle platforms generally suffer from data update lags and information loss across stages. Synchronizing detection results with BIM models still requires significant manual effort, and the absence of an automated data pipeline severely constrains closed-loop data-to-decision capabilities [26,139].

### 4.2. Future Research Perspectives

Advancing building façade pathology inspection requires a shift from fragmented optimizations to a full-process collaborative system. Despite progress in path planning, multi-modal fusion, recognition algorithms, and digital twins, current research lacks adaptability to complex environments, cross-scene generalization, and efficient real-time edge processing. Future research should focus on four key areas: autonomous cognitive planning, generalized and lightweight data fusion, precision-efficiency synergistic recognition, and lifecycle digital twin. This approach aims to transition from isolated technological advancements to comprehensive synergistic optimization, guiding the intelligent development of urban building safety O&M systems.

#### 4.2.1. Evolution of Path Planning Toward Autonomous Cognition

Future research should drive UAV inspection path planning toward an autonomous cognitive framework, moving beyond reliance on offline BIM models or preset rules. By integrating DL, GNN, and reinforcement learning, lightweight online semantic mapping and real-time decision loops can achieve “perceive-as-you-fly, optimize-as-you-perceive” planning [34,35,38,41]. Tight-coupling fusion of vision, LiDAR, IMU, and UWB must be strengthened to ensure robust centimeter-level localization and environmental modeling under GNSS-denied or dynamically interfering conditions [18,39,40]. Ultimately, scene-adaptive strategies that dynamically adjust viewpoint density and sensing modalities based on façade complexity and weather conditions will drive the transition from tool-assisted to fully autonomous intelligent inspection [41].

#### 4.2.2. Toward Generalized and Lightweight Multi-Modal Fusion Frameworks

To address the scene/sensor specificity and edge deployment bottlenecks of existing fusion methods, future research should construct generalized yet lightweight multi-modal fusion frameworks. For generalization, self-supervised and contrastive learning can extract modality-agnostic features, while domain generalization and meta-learning enhance cross-domain adaptability to unseen scenes and few-shot tasks [44,49,56,59,60,62]. For lightweight design, Neural Architecture Search and Knowledge Distillation, which combined with quantization, operator fusion, and hardware-aware pruning, are needed to transfer complex network capabilities to onboard platforms [22,52,140,141]. Finally, a task-driven end-to-end joint optimization mechanism should eliminate computational redundancy between fusion and downstream recognition, achieving precision–real-time synergy under resource constraints [44,57].

#### 4.2.3. Precision-Efficiency Synergistic Recognition Algorithms

To move beyond the precision–efficiency trade-off, future recognition algorithms should adopt a synergistic lightweight design [64,113]. NAS can discover Pareto-optimal structures under specific UAV hardware constraints. Dynamic inference mechanisms—adjusting computational depth or channel width based on real-time scene complexity—enable refined segmentation for complex defects and high-speed processing for simple backgrounds [140,141]. A cloud-edge collaborative architecture can further compensate for onboard limits through edge-side preliminary screening and cloud-based refinement [142]. For training, structured knowledge distillation of fine-grained representations, combined with self-supervised pre-training on unlabeled data, will significantly improve both accuracy and inference speed on resource-constrained platforms [17].

#### 4.2.4. Lifecycle Digital Twin O&M System Construction

**Figure 5:**
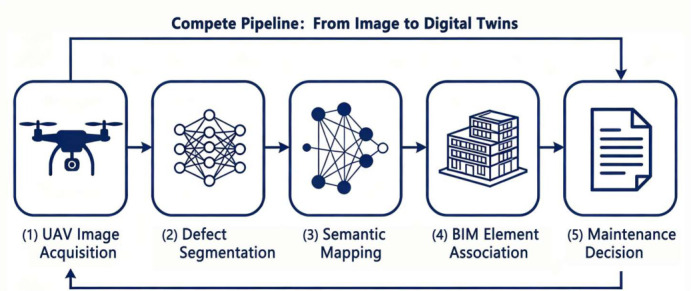
The complete pipeline of the building’s lifecycle O&M system.

Future digital twin development for building O&M should shift from isolated technical optimization to full-chain collaboration, prioritizing 3D defect mapping, BIM integration, and maintenance decision-making (Figure 5). For 3D defect mapping, fusing GPS, visual data, and depth registration enables millimeter-level projection of 2D defect pixels onto 3D model space, requiring a quantitative error propagation model to evaluate localization uncertainty [36,129,132,134]. For BIM component linking, standardized semantic descriptions (e.g., IFC property sets or ontologies) are needed to automatically map deep learning masks to building components, while ICDD-based multi-model integration records component state evolution from temporal inspection data [136,137,138]. For component state updating, long-term inspection data should support temporal correlation and deterioration trend analysis. The ICDD-based multi-model integration approach standardizes the fusion of geometric models, semantic information, and dynamic monitoring data, enabling component state evolution recording [137]. For maintenance decision-making, defect assessment should leverage periodic inspection databases. For instance, the placeholder family by Matos et al. [28] prioritizes repairs for lightweight O&M. Maintenance recommendations should combine DL models with local/national standards, automatically matching standardized repair suggestions according to the assessment [137,139].

## 5. Conclusions

As urban building management shifts toward existing inventories, traditional manual inspections are increasingly inadequate for façade safety efficiency. The integration of UAVs and DL is driving a paradigm shift in façade pathology inspection, which has shifted from sporadic manual sampling to a smart, automated framework. Based on a systematic review of 135 papers across four key technical domains, this section addresses research questions Q1–Q4 from Section 2.1. The responses are as follows.

In response to Q1, autonomous UAV flight path planning in complex high-density urban environments, the real-time SLAM and online viewpoint generation strategy for “mapping while flying” incurs high computational overhead, limiting deployment on resource-constrained onboard platforms and keeping it experimental. In contrast, edge-based lightweight deep learning for real-time obstacle recognition and path replanning is more mature. Although it sacrifices global planning due to limited onboard resources, external modules (e.g., SLAM) can supplement this, enhancing practical deployment potential. BIM-dependent offline planning suits new buildings with complete digital documentation. Current deployable approaches include elliptical spiral trajectories and BIM-integrated edge computing. Overall, the “perception–mapping–decision” coordination mechanism requires further real-world validation, as existing studies remain largely at the laboratory simulation stage.

In response to Q2, deep fusion of multi-source heterogeneous data requires three sequential stages: spatial alignment, adaptive fusion, and 3D fusion. For spatial alignment, edge extremal point and SIFT-based registration methods are well established but lack robustness in low-texture scenes. Deep learning approaches like SuperGlue achieve high accuracy at the cost of substantial computation and remain experimental. In adaptive fusion, feature-level networks such as LBF^2^Net offer superior performance but have large parameter counts that hinder edge deployment; lightweight architectures like MobileNet and knowledge distillation are more feasible. Generative adversarial networks and modality hallucination methods still need validation for training stability and generalization. Integrating multi-modal fusion with 3D semantic mapping of defects remains an open direction requiring further exploration for engineering implementation.

In response to Q3, current deep learning approaches for multiple façade defect recognition exhibit a clear task–architecture co-adaptation rather than relying on a single universal model. For fine-grained crack detection, USSA-Net and LGFAF-Net achieve superior segmentation accuracy on public datasets like DeepCrack. Attention-enhanced single-stage detectors, such as MAE-YOLOv8, offer a practical trade-off between accuracy and efficiency for real-time detection of defects like spalling and water seepage. Multi-modal architectures (e.g., visible–thermal cascading) show promise for concealed defects like delamination, but their generalization and real-time performance remain unverified at the engineering level. For UAV edge deployment, ultra-lightweight models like UMDA maintain high accuracy even under severe parameter compression, as validated by onboard inspection. Thus, algorithm selection should be task-specifically tailored rather than seeking a universal architecture.

In response to Q4, integrating UAV defect recognition outputs into BIM involves three stages: spatial registration, defect semantic mapping, and twin integration with time-series monitoring data. Spatial registration, which fuses GPS, visual data, and depth maps for millimeter-level 3D localization of 2D defect pixels, is relatively mature with preliminary engineering applications. Semantic mapping, using IFC property sets or ontology-based defect frameworks, remains experimental; automating the association between deep learning output masks and BIM components is not yet standardized, resulting in low automation and representing the primary technical bottleneck. For twin integration, single-platform BIM-centric approaches have extensive engineering practice. Although ICDD-based multi-model integration offers significant advantages, its data exchange efficiency and real-time performance currently fall short of engineering requirements, necessitating deployment trials in real O&M scenarios. The goal is integration of geometric, semantic, and temporal data to enable a complete closed loop from inspection to O&M decisions, establishing a digital twin O&M platform across the full building lifecycle.

In summary, the convergence of UAV and DL technologies is transforming urban building inspection. As advances in autonomous perception, adaptive fusion, precision-efficiency recognition, and dynamic digital twins emerge, future intelligent inspection systems will evolve from auxiliary tools into fully autonomous, high-reliability engineering-grade intelligent agents. These developments will provide a robust technical foundation for the refined management and intelligent O&M of the urban built environment.

## Figures and Tables

**Table 1:** Categorization of research objects in the selected literature.

Categories of Research Objects		Paper Count
Building	Façade	39
	Structure	39
Concrete		39
Post-Disaster Building		12
Infrastructure	Hydraulic	2
	Electricity	1
	Underground	2
	Comprehensive	17
Bridge		20
Asphalt Pavement		10
Cultural Heritage		7
Other Scenarios		4

**Table 2:** Journals in which the shortlisted papers were published.

Journal	Category	Count	Percentage (%)	Impact Factor
*Sensors*	Instruments and Instrumentation	14	10.1	3.7
*Automation in Construction*	Civil Engineering	12	8.7	12.7
*Journal of Building Engineering*	Civil Engineering	8	5.8	7.5
*IEEE Access*	Computer Science	7	5.1	3.9
*Drones*	Remote Sensing	6	4.4	5.0
*Buildings*	Civil Engineering	5	3.6	3.2
*Advanced Engineering Informatics*	Artificial Intelligence	4	2.9	10.0
*Measurement*	Instrumentation	3	2.2	5.4
*Engineering Structures*	Engineering	3	2.2	6.3
*Applied Sciences-Basel*	Engineering, Multidisciplinary	3	2.2	2.7
*IEEE Sensors Journal*	Engineering	3	2.2	4.7
*Journal of Computing in Civil Engineering*	Civil Engineering	3	2.2	6.5
*Journal of Performance of Constructed Facilities*	Civil Engineering	3	2.2	2.5
*Construction and Building Materials*	Construction and Building Technology	2	1.45	8.6
*Engineering Applications of Artificial Intelligence*	Artificial Intelligence	2	1.45	7.7
*Electronics*	Computer Science	2	1.45	2.6
*Infrastructures*	Construction and Building Technology	2	1.45	3.0
*IEEE Transactions on Automation Science and Engineering*	Automation and Control Systems	2	1.45	7.0
*IEEE Transactions on Instrumentation and Measurement*	Instruments and Instrumentation	2	1.45	6.0
*Journal of Civil Structural Health Monitoring*	Civil Engineering	2	1.45	4.4
*Earthquake Engineering and Engineering Vibration*	Civil Engineering	2	1.45	3.3
*Structural Control & Health Monitoring*	Civil Engineering	2	1.45	7.3
*Frontiers in Built Environment*	Civil Engineering	2	1.45	2.9
*Scientific Reports*	Science and Technology	2	1.45	4.3

**Table 3:** Comparison of UAV inspection path planning methods.

Reference	Required Input Model	GPS Dependence	Obstacle Avoidance	Suitability for High-Rise Façades	Computational Cost	Technical Maturity
Zhao et al. [34]	Pre-built 3D mesh model	Weak; relies more on the pre-built geometric model than on GPS	Only model-based offline obstacle avoidance	Primarily designed for bridges	High; requires two-stage algorithmic optimization	Laboratory validation stage
Tong et al. [35]	Pre-built 3D mesh model	Not explicitly discussed	Only offline obstacle avoidance for the target structure itself	Primarily designed for high-rise buildings	High; stage-wise computation	Laboratory validation stage
Tan et al. [26]	Pre-built 3D mesh model (BIM)	Highly dependent on GPS/RTK	Only model-based offline obstacle avoidance	Primarily designed for high-rise buildings	High; controlled by stage-wise computation	Deployment stage
Mathur et al. [36]	No 3D model required	Not explicitly discussed	Online active obstacle avoidance capability	Primarily designed for high-rise buildings	High; real-time trajectory optimization, crack detection, and 3D reconstruction	Laboratory validation stage,
Cui et al. [18]	No 3D model required (generated by LLM analyzing)	Relies on GPS positioning	Online active obstacle avoidance capability	Primarily designed for transmission lines and photovoltaic power stations	High; requires GPU acceleration for LLM and high-performance ground station or onboard edge computing	Deployment stage
Waqas et al. [40]	No 3D model required	Completely independent of GPS	Online active obstacle avoidance capability	Primarily designed for horizontal or low-altitude structures	Moderate	Deployment stage
Song et al. [27]	No 3D model required (builds model via online MVS)	Completely independent of GPS	Only obstacle avoidance in static environments	Primarily designed for large-scale structures	High; high-performance GPU and CPU, for real-time performance	Deployment stage
Wang et al. [38]	Pre-built 3D mesh model (BIM)	Highly dependent on GPS/RTK	Only model-based offline obstacle avoidance	Primarily designed for bridge	Low; planning based on geometric calculations and heuristic rules (non-iterative)	Deployment stage
Liu et al. [41]	Pre-built 3D mesh model (IFC/BIM)	Relies on GPS or global positioning system	Only offline static obstacle avoidance and occlusion handling	Based on 3D mesh coverage; discretizes surfaces into planar/non-planar meshes	High; computational load increases with number of viewpoints	Laboratory validation stage

**Table 4:** Comparison of representative multi-modal fusion methods.

Reference	Defect Type	Sensor Modality	Fusion Method	Advantages	Limitations
Yang et al. [49]	Detached and missing tiles (building façade)	RGB and Thermal	Based on U-Net, comparison of early, intermediate, and late fusion for façade defect detection	Early fusion: highest mAP Intermediate fusion: best parameter efficiency Late fusion: sensitive to specific defects	Performance saturation/degradation with increased model depth Late fusion: hyperparameter-sensitive, prone to collapse
Adriano et al. [50]	Post-disaster building collapse/damage	Optical Imagery and SAR	U-Net for semantic segmentation; five pre- and post-disaster data modality scenarios for performance comparison	Pre-optical + post-SAR enables operation under adverse weather Cross-modality effectively compensates for missing optical data	SAR-only performance drops significantly (mean F1 ~0.25) Low accuracy for “moderate damage” level
Pozzer et al. [51]	Crack, spalling, potential subsurface defect (concrete)	RGB and Thermal	Comparison of single-modality vs. fused images on CNN segmentation performance	Fused images cover more defect classes without significant information loss Image enhancement increases entropy	Crack segmentation IoU remains generally low (0.30–0.40) Fusion not superior to the best single modality
He et al. [52]	Delamination, water infiltration, spalling (building façade)	RGB and Thermal	the Layered Bilateral Feature Fusion Network (LBF2-Net): a complementary attention branch and a consistent attention branch	IoU: 0.8156, F1: 0.7819, outperforming BBS-Net Explicitly distinguishes surface defects from subsurface defects Validated on real campus building façades	Uneven distribution of defect types in dataset IR image shadows/reflections may cause false positives
Yuan et al. [22]	Crack (asphalt pavement, brick, and stone walls)	RGB Thermal	a structure-aware progressive multi-modal fusion network (SPMFNet) for RGB-thermal (RGB-T) crack segmentation	mIoU: 0.861, F1: 0.854 on asphalt and masonry crack datasets Edge guidance improves fine-crack continuity	Relatively high model complexity
Hussain et al. [54]	Crack (concrete structure)	RGB and Depth	RGB-D crack segmentation network, CSANet: using a double cross-fusion module to integrates Depth features with RGB	4.54% mIoU gain over RGB-only via depth fusion Depth enables pixel-to-mm spatial calibration Crack width absolute error < 0.05 mm	Depth quality affected by ambient light Fine crack boundary detection needs improvement
Wang et al. [46]	Leakage, efflorescence, crack, hollow (building façade)	RGB and Thermal	End-to-end method by integrating multi-modal image registration, infrared–visible image fusion (IVIF), and damage segmentation	RMSE: 14.35, outperforming RIFT and others Instance segmentation mAP: 85.4%	IVIF module may miss weak-contrast damage features
Ren et al. [57]	Crack (concrete structures)	RGB and Depth	SRFormer + SQFormer (Self-Query Transformer + IFFB)	SR + multi-modal fusion achieves best performance (F1: 92.29%, mIoU: 90.62%) Detects 0.1 mm thin cracks, width error < 0.02 mm	High computational cost and long processing time for SR images False positives under extreme low light or crack-like depth textures
Mondal and Jahanshahi [59]	Spalling, exposed rebars, buckled rebars (concrete structure)	RGB and Depth	An autonomous damage segmentation framework based on hallucination (MH) and monocular depth estimation (MDE)	No depth sensor needed at test time, saving cost MH IoU: 0.874–0.891, close to real RGB-D, with fast processing	Relies on synthetic data, lacks real-world validation MDE method is computationally heavy and slow
Liu et al. [44]	Crack (asphalt pavement)	RGB and Thermal	A novel multi-modal crack segmentation method (FC_SAM), utilizing fine-tuned large vision models.	Only 300 training samples achieve IoU 86.41% Strong cross-device generalization (IoU 64.29%) Parameter-efficient fine-tuning	Large model (639M params), inference at 5.35 FPS Potential under-detection at extremely fine crack tips

**Table 5:** The statistics of the defect type in the references of Section 3.3.

Defect Type	Reference Count	Reference
Crack	55	[5,14,16,17,46,63,64,65,66,67,68,69,70,71,72,73,74,75,76,77,78,79,80,81,82,83,84,85,86,87,88,89,90,91,92,93,94,95,96,97,98,99,100,101,102,103,104,105,106,107,108,109,110,111,112]
Spalling	17	[5,63,65,66,67,71,72,77,79,90,92,96,98,102,103,105,113]
Corrosion	10	[63,65,66,67,71,72,87,90,92,114]
Rebar Exposure	6	[14,63,77,92,98,115]
Leakage	8	[5,17,46,65,67,79,105,116]
Efflorescence	6	[14,46,63,65,92,103]
Bulge	2	[46,67]

**Table 6:** Performance comparison of different crack semantic segmentation models on the DeepCrack dataset.

Model	mIoU (%)	F1-Score (%)	Precision (%)	Recall (%)	Inference Hardware	Image Resolutions	Train/Test Protocols	Evaluation Indicators
UMDA	87.0	82.5	-	85.7	NVIDIA Tesla M40 GPU	480 × 480 (resize)	Training sets: 477 Test Sets: 60	F1-Score IOU MIOU Recall
U-Net	83.3	77.8	-	77.3				
DeepLabV3	76.2	74.2	-	82.9				
DeepLabV3*	81.0	64.9	-	84.4				
LR-ASPP	82.7	75.2	-	76.6				
EfficientNet	81.5	75.8	-	78.4				
ShuffleNetV2	80.7	77.4	-	79.3				
MobileVIT	80.3	75.1	-	80.1				
CrackSegNet	82.7	82.1	-	81.7				
CrackFormer	81.8	81.7	-	81.5				
CT-CrackSeg	86.2	84.1	-	84.0				
ABANet	85.7	81.4	-	80.7				
LGFAF-Net	89.4	88.7	89.8	87.5	NVIDIA GTX 3090 GPU	544 × 384 (original size)	Training sets: 300 Test Sets: 237	Precision Recall F1-Score mIoU
CrackSegNet	87.5	86.4	85.7	87.1				
EMRA-Net	88.1	87.1	88.0	86.3				
CrackFormer-II	87.5	86.4	84.9	88.0				
APFNet	87.8	86.7	84.7	88.9				
CrackSight	81.7	89.9	91.3	88.6	NVIDIA A100 GPU	448 × 448 (resize)	Training Sets: 376 Validation Sets: 107 Test Sets: 54	Precision Recall F1-Score mIoU
DPCT-Net	87.0	85.7	89.3	82.3				
DECS-Net	75.2	87.5	82.9	92.7				
GGMNet	77.2	87.1	83.6	90.9				
CrackFormer	75.7	86.2	81.2	91.8				
CCDFormer	76.1	86.5	88.5	84.6				
U-Net	72.9	84.4	79.2	90.3				
TransUNet	72.5	84.0	78.0	91.0				
DeepCrack	71.8	83.6	79.6	87.9				
DeepLabV3+	70.6	82.8	75.8	91.2				
SegNet	71.9	83.6	79.4	88.3				

**Table 7:** Comparison of lightweight crack detection models and their edge deployment characteristics.

Reference	Model	Size	Edge Hardware	FPS	Latency	Flight Conditions	Performance Metrics
Han et al. [64]	UMDA	40 MB	Intel i3-8100 CPU	61.2	16.34 ms/Image (480 × 480)	Indoor laboratory environment, Not deployed on UAV	DeepCrack: IOU: 75.3% mIoU: 87.0% Recall: 85.7%
Gonthina et al. [70]	CrackScan	-	Raspberry Pi 4			Actual UAV flight test (field test)	Crack500: mIoU: 82.5%
Altaf et al. [68]	MobileVNet	3.2 MB	The platform for discussion: NVIDIA Jetson Nano, Raspberry Pi 4 Actual testing: Google Colab (GPU)	83	12 ms/Image	Not deployed on UAV	Accuracy: 89.45%
	RSNet	6.5 MB		56	18 ms/Image		Accuracy: 90.6%
	CNN-Simple	1.8 MB		100	10 ms/Image		Accuracy: 87.22%
Huang et al. [75]	CladdingNet	-	NVIDIA Jetson AGX Xavier (onboard)	1.96	260 ms/Image	Actual UAV flight test (field test) velocity:0.5 m/s Maximum wind velocity: 15 m/s	mIoU (Focal Loss): 0.76 mIoU (Cross- Entropy Loss): 0.79
Kumar et al. [102]	Tiny YOLOv3	34 MB	NVIDIA Jetson TX2 (onboard)	5.3	188 ms/Image	Indoor laboratory environment	Precision: 0.94 Recall: 0.89 F1: 0.91

## Data Availability

These data were derived from the following resources available in the public domain: [Web of Science, https://webofscience-clarivate-cn.accproxy.lib.szu.edu.cn/wos/woscc/basic-search (accessed on 19 June 2026)].

## Nomenclature

BIM	Building Information Modeling
BLM	Building Lifecycle Management
CAD	Computer-Aided Design
CAM	Class Activation Mapping
CG	Computer Graphics
CGAN	Conditional Generative Adversarial Network
CNN	Convolutional Neural Network
DCGAN	Deep Convolutional Generative Adversarial Network
DFT	Discrete Fourier Transform
DL	Deep Learning
DOD	Degree of Damage
DT	Digital Twin
ECEF	Earth-Centered, Earth-Fixed
FEM	Finite Element Model
FoV	Field of View
GA	Genetic Algorithm
GAN	Generative Adversarial Network
GCA	Gated Control Attention
GIS	Geographic Information System
GNSS	Global Navigation Satellite System
GNN	Graph Neural Network
GPR	Ground Penetrating Radar
HBIM	Historic Building Information Modeling
ICDD	Information Container for Linked Document Delivery
IFC	Industry Foundation Classes
IMU	Inertial Measurement Unit
LiDAR	Light Detection and Ranging
LIFT	Local Intensity Binary Transformation
LLM	Large Language Model
LOD	Level of Detail
mAP	mean Average Precision
mIoU	mean Intersection over Union
ML-ADTSP	Multi-Layer Angle-Distance Traveling Salesman Problem
MVS	Multi-View Stereo
NAS	Neural Architecture Search
NEZ	Northeast Sky
NMS	Non-Maximum Suppression
O&M	Operation and Maintenance
OWL	Web Ontology Language
PBGM	Physics-Based Graphics Model
PnP	Perspective-n-Point
PPM	Pyramid Pooling Module
RGB-D	Red, Green, Blue—Depth
RMSE	Root Mean Square Error
RTK	Real-Time Kinematic
SAR	Synthetic Aperture Radar
SfM	Structure from Motion
SHACL	Shapes Constraint Language
SLAM	Simultaneous Localization and Mapping
SNN	Siamese Neural Network
SRFBN	Super-Resolution Feedback Network
SRR	Super-Resolution Reconstruction
TOP	Team Orienteering Problem
UAV	Unmanned Aerial Vehicle
UBS	Ultrasonic Beacon System
UWB	Ultra-Wideband
ViT	Vision Transformer
WGS84	World Geodetic System 1984
WoS	Web of Science

## References

[1] Farahzadi L., Odeh I., Kioumarsi M., Shafei B. (2025). Automated image-based condition assessment of the built environment: A state-of-the-art investigation of damage characteristics and detection requirements. Results Eng..

[2] Wang P., Xiao J., Qiang X., Xiao R., Liu Y., Sun C., Hu J., Liu S. (2024). An automatic building façade deterioration detection system using infrared-visible image fusion and deep learning. J. Build. Eng..

[3] Ma F., Zhang D., Wang Z., Chen X., Jiang L. (2023). Risk Assessment of Falling Objects from Façades of Existing Buildings. Buildings.

[4] Rakha T., Gorodetsky A. (2018). Review of Unmanned Aerial System (UAS) applications in the built environment: Towards automated building inspection procedures using drones. Autom. Constr..

[5] Zhou X.L., Tiong R.L.K. (2026). Defects inspection system for building facades using drones and deep learning method. Expert Syst. Appl..

[6] Jeong K., Kwon J., Do S.L., Lee D., Kim S. (2022). A Synthetic Review of UAS-Based Facility Condition Monitoring. Drones.

[7] Lyu C., Lin S.Q., Lynch A., Zou Y., Liarokapis M. (2025). UAV-based deep learning applications for automated inspection of civil infrastructure. Autom. Constr..

[8] Gohari A., Bin Ahmad A., Rahim R.B., Supa’at A.S.M., Abd Razak S., Gismalla M.S.M. (2022). Involvement of Surveillance Drones in Smart Cities: A Systematic Review. IEEE Access.

[9] Perera P., Perera S., Jin X.H., Rashidi M., Nanayakkara S., Yazbek G., Yazbek A. (2025). Deep learning—Enabled visual computing in construction: Application and digital technology integration. Front. Built Environ..

[10] Zhuang H.Y., Cheng Y.K., Zhou M., Yang Z.J. (2025). Deep learning for surface crack detection in civil engineering: A comprehensive review. Measurement.

[11] Pan X., Yang T.T.Y., Li J., Ventura C., Málaga-Chuquitaype C., Li C.B., Su R.K.L., Brzev S. (2025). A review of recent advances in data-driven computer vision methods for structural damage evaluation: Algorithms, applications, challenges, and future opportunities. Arch. Comput. Methods Eng..

[12] Cha Y.J., Ali R., Lewis J., Büyükoztürk O. (2024). Deep learning-based structural health monitoring. Autom. Constr..

[13] Sony S., Dunphy K., Sadhu A., Capretz M. (2021). A systematic review of convolutional neural network-based structural condition assessment techniques. Eng. Struct..

[14] Cabral R., Santos R., Correia J., Ribeiro D. (2025). A Hybrid YOLO and Segment Anything Model Pipeline for Multi-Damage Segmentation in UAV Inspection Imagery. Sensors.

[15] Silva A.S., Neto F., Ferreira P.H., Costa D.B. (2025). CNN-Based YOLOv12 for Damage Assessment in Residential Roofs. IEEE Access.

[16] Gan L.F., Liu H., Yan Y., Chen A.R. (2024). Bridge bottom crack detection and modeling based on faster R-CNN and BIM. IET Image Process..

[17] Fan Y., Mai J.H., Xue F., Lau S.S.Y., Jiang S., Tao Y.Q., Zhang X.X., Tsang W.C. (2025). UAV and Deep Learning for Automated Detection and Visualization of Façade Defects in Existing Residential Buildings. Sensors.

[18] Cui L.N., Zhou K.H., Wang J.H., Du Z., Jiang C.Y., Qin H. (2025). Research on UAV swarm inspection path and defect identification based on LLM multi-agent collaborative optimization. Microchem. J..

[19] Zheng J., Tan J., Chen G. (2025). Research on UAV coverage path planning in building visual inspection. J. Build. Eng..

[20] Fayyad T.M., Taylor S., Feng K., Hui F.K.P. (2025). A scientometric analysis of drone-based structural health monitoring and new technologies. Adv. Struct. Eng..

[21] Martin M., Chong A.D., Biljecki F., Miller C. (2022). Infrared thermography in the built environment: A multi-scale review. Renew. Sustain. Energy Rev..

[22] Yuan Z.R., Ding X., Xia X.H., He Y.B., Fang H., Yang B., Fu W. (2025). Structure-Aware Progressive Multi-Modal Fusion Network for RGB-T Crack Segmentation. J. Imaging.

[23] Elias M., Weitkamp A., Eltner A. (2023). Multi-modal image matching to colorize a SLAM based point cloud with arbitrary data from a thermal camera. Isprs Open J. Photogramm. Remote Sens..

[24] Hai L., Wang B.R., Zhang T.T., Guo W.L., Zhang Q.Q., Ma Z.X. (2025). Multi-modal sensing data fusion for bridge displacement estimation: A review. Intell. Transp. Infrastruct..

[25] Mardanshahi A., Sreekumar A., Yang X., Barman S.K., Chronopoulos D. (2025). Sensing Techniques for Structural Health Monitoring: A State-of-the-Art Review on Performance Criteria and New-Generation Technologies. Sensors.

[26] Tan Y., Yi W., Chen P.L., Zou Y. (2024). An adaptive crack inspection method for building surface based on BIM, UAV and edge computing. Autom. Constr..

[27] Song S., Kim D., Choi S. (2022). View Path Planning via Online Multiview Stereo for 3-D Modeling of Large-Scale Structures. IEEE Trans. Robot..

[28] Matos R., Rodrigues H., Costa A., Rodrigues F. (2023). BIM-FM integrated solution resourcing to digital techniques. Neural Comput. Appl..

[29] Plevris V., Papazafeiropoulos G. (2024). AI in Structural Health Monitoring for Infrastructure Maintenance and Safety. Infrastructures.

[30] Ferraris C., Amprimo G., Pettiti G. (2023). Computer Vision and Image Processing in Structural Health Monitoring: Overview of Recent Applications. Signals.

[31] Forlesi M., Esposito A., Zyrianoff I., Marzani A., Leonardi G., Di Felice M. (2025). Crack Detection and Monitoring: Review and Comparison of IoT and Image-Based Methods Roadmap for Measurement and Applications. IEEE Instrum. Meas. Mag..

[32] Bolourian N., Hammad A. (2020). LiDAR-equipped UAV path planning considering potential locations of defects for bridge inspection. Autom. Constr..

[33] Woo H.J., Hong W.H., Oh J., Baek S.C. (2023). Defining Structural Cracks in Exterior Walls of Concrete Buildings Using an Unmanned Aerial Vehicle. Drones.

[34] Zhao Y.X., Lu B.H., Alipour M. (2024). Optimized structural inspection path planning for automated unmanned aerial systems. Autom. Constr..

[35] Tong H.W., Li B.Y., Huang H.L., Wen C.Y. (2025). Multi-Layer Path Planning for Complete Structural Inspection Using UAV. Drones.

[36] Mathur P., Sharma C., Azeemuddin S. (2024). Autonomous Inspection of High-Rise Buildings for Façade Detection and 3D Modeling Using UAVs. IEEE Access.

[37] Hu D., Li S., Du J., Cai J.N. (2023). Automating Building Damage Reconnaissance to Optimize Drone Mission Planning for Disaster Response. J. Comput. Civ. Eng..

[38] Wang F., Zou Y., Castillo E.D., Ding Y.L., Xu Z., Zhao H.W., Lim J.B.P. (2024). Automated UAV path-planning for high-quality photogrammetric 3D bridge reconstruction. Struct. Infrastruct. Eng..

[39] Tse K.W., Pi R.D., Sun Y.X., Wen C.Y., Feng Y.R. (2023). A Novel Real-Time Autonomous Crack Inspection System Based on Unmanned Aerial Vehicles. Sensors.

[40] Waqas A., Kang D.H., Cha Y.J. (2024). Deep learning-based obstacle-avoiding autonomous UAVs with fiducial marker-based localization for structural health monitoring. Struct. Health Monit..

[41] Liu J.C., Li H.J., Wang D.L., Chai C.Z., Dong Y.Q. (2025). Semantic-PolygonGraph driven context-aware coverage path planning for infrastructure visual inspection. Adv. Eng. Inform..

[42] Zeng J.C., Wu Z.H., Todd M.D., Hu Z. (2023). Bayes risk-based mission planning of Unmanned Aerial Vehicles for autonomous damage inspection. Mech. Syst. Signal Process..

[43] Ali R., Kang D.H., Suh G., Cha Y.J. (2021). Real-time multiple damage mapping using autonomous UAV and deep faster region-based neural networks for GPS-denied structures. Autom. Constr..

[44] Liu Z.X., Wang J.J., Teng X.Y., Li N. (2025). Fine-tuning large vision model for multimodal fusion in asphalt pavement crack segmentation. Int. J. Pavement Eng..

[45] Lin D., Yang N., Miao Q., Cui X.J., Xu D.G. (2025). True 3D thermal inspection of buildings using multimodal UAV images. J. Build. Eng..

[46] Wang P.J., Wang J.H., Liu Q., Fang L., Xiao J. (2025). Fusion-Based Damage Segmentation for Multimodal Building Façade Images from an End-to-End Perspective. Buildings.

[47] Shahsavarani S., Lopez F., Ibarra-Castanedo C., Maldague X.P.V. (2024). Robust Multi-Modal Image Registration for Image Fusion Enhancement in Infrastructure Inspection. Sensors.

[48] Shahsavarani S., Lopez F., Ibarra-Castanedo C., Maldague X.P.V. (2024). Advanced Image Stitching Method for Dual-Sensor Inspection. Sensors.

[49] Yang X.C., Guo R.H., Li H. (2023). Comparison of multimodal RGB-thermal fusion techniques for exterior wall multi-defect detection. J. Infrastruct. Intell. Resil..

[50] Adriano B., Yokoya N., Xia J.S., Miura H., Liu W., Matsuoka M., Koshimura S. (2021). Learning from multimodal and multitemporal earth observation data for building damage mapping. Isprs J. Photogramm. Remote Sens..

[51] Pozzer S., De Souza M.P.V., Hena B., Hesam S., Rezayiye R.K., Azar E.R., Lopez F., Maldague X. (2022). Effect of different imaging modalities on the performance of a CNN: An experimental study on damage segmentation in infrared, visible, and fused images of concrete structures. NDT E Int..

[52] He S.D., Zhang S.H., Mishra D., Yuen M.M.F. (2026). Layered Bilateral Feature Fusion Network for end-to-end defect segmentation on aging tiled building Façades. Adv. Eng. Inform..

[53] Zhao Y.J., Lai H.C., Gao G.X. (2023). RMFNet: Redetection Multimodal Fusion Network for RGBT Tracking. Appl. Sci..

[54] Hussain T., Li Y.C., Ren M.Y., Li J.C. (2025). Pixel-level crack segmentation and quantification enabled by multi-modality cross-fusion of RGB and depth images. Constr. Build. Mater..

[55] Wang F.Z., Huang J.Z., Fu Y. (2025). Convolutional neural network-based multimodal image information fusion for moisture damage assessment of cultural heritage buildings. Measurement.

[56] Roheda S., Krim H., Riggan B.S. (2021). Robust Multi-Modal Sensor Fusion: An Adversarial Approach. IEEE Sens. J..

[57] Ren M.Y., Li Y.C., Hussain T., Wu Y.J., Li J.C. (2026). Pixel-level concrete crack quantification through super resolution reconstruction and multi-modality fusion. Adv. Eng. Inform..

[58] Su S., Yan L., Zhou Y.Q., Wang P.Z., Chen C.J. (2025). Visible and Infrared Image Fusion Based on Modality Feature Enhancement for Localization in Low-Light Environments. IEEE Sens. J..

[59] Mondal T.G., Jahanshahi M.R. (2023). Fusion of color and hallucinated depth features for enhanced multimodal deep learning-based damage segmentation. Earthq. Eng. Eng. Vib..

[60] Yu Y., Kang S., He D.Q., Kumar R., Singh V., Wang Z.F. (2025). Comparison of Asphalt Pavement Crack Segmentation Based on Different Fusion Methods of RGB Images and Thermal Images. J. Transp. Eng. Part B Pavements.

[61] Chen Y.W., Chen X., Chen B.M. (2026). Enhanced building thermal defect detection using deep learning-based multimodal fusion based thermographic reconstruction. J. Build. Eng..

[62] Pozzer S., Ramos G., Azar E.R., Osman A., El Refai A., López F., Ibarra-Castanedo C., Maldague X. (2024). Enhancing concrete defect segmentation using multimodal data and Siamese Neural Networks. Autom. Constr..

[63] Bae H., Cho Y., An Y.K. (2025). Scale-adaptive refinement module-based deep segmentation network for automated evaluation of multi-type damage on concrete buildings. J. Build. Eng..

[64] Han B.L., Zhang Y.H., Huang C.Y., Ding W., Liu Z.W., Deng H.Y., Wu J. (2026). UMDA: Lightweight and Efficient Crack Segmentation Model. J. Comput. Civ. Eng..

[65] Meng B.X., Wang C.Q. (2026). Drone-Guided Cognitive Feature Aggregation for High-Rise Building Surface Defect Inspection. IEEE Access.

[66] Wang J.H., Yang Y., Wang Z.L., Lv L.Y., Ueda T. (2026). A scalable UAV-based structural health monitoring framework using augmented deep learning for multilevel damage classification. J. Civ. Struct. Health Monit..

[67] Zha Q.K., Yao Y.M., Zheng Y.F., Ma W.Q., Zhang W.K. (2025). A dataset of building surface defects collected by UAVs for machine learning-based detection. Sci. Data.

[68] Altaf A., Mehmood A., Filograno M.L., Alharbi S., Iqbal J. (2025). Deployable Deep Learning Models for Crack Detection: Efficiency, Interpretability, and Severity Estimation. Buildings.

[69] Amirkhani D., Allili M.S., Lapointe J.F. (2025). CrackSight: An Efficient Crack Segmentation Model in Varying Acquisition Ranges and Complex Backgrounds. IEEE Trans. Autom. Sci. Eng..

[70] Gonthina S.S., Aditya S.V.S., Gannoju A.T., Das D., Sinha A. (2025). CrackScan: Enabling Intelligent Edge Inspection with UAVs for Structural Health Monitoring. IEEE Sens. J..

[71] Huang L.J., Fan G., Li J., Chen S.H., Peng Z., Hao H. (2025). Automated surface damage detection and quantification in concrete structures using semantic point clouds. Eng. Struct..

[72] Meda D., Ahmed M.M., Kalapatapu P., Pasupuleti V.D.K. (2025). Enhanced Structural Damage Detection, Segmentation, and Quantification Using Computer Vision and Deep Learning. J. Comput. Civ. Eng..

[73] Silva C., Dissanayake D., Rathnayake R., Sathsara N. (2025). Aerial crack detection in building structures using convolutional neural networks. J. Natl. Sci. Found. Sri Lanka.

[74] Yogi B., Das S.K., Modak S., Biswas A., Roy S. (2025). Early surface crack detection and localization in structures: An artificial intelligence approach. Discov. Appl. Sci..

[75] Huang B.F., Shi J.W., Guan X.Q., Liu X.Z., Yao X.T., Liu J.H. (2025). An unmanned aerial vehicle implemented network for real-time crack detection in stone cladding. Comput.-Aided Civ. Infrastruct. Eng..

[76] Lethanh N., Trinh T.A., Hossain M.T. (2025). An Investigation on Prediction of Infrastructure Asset Defect with CNN and ViT Algorithms. Infrastructures.

[77] Tauzowski P., Ostrowski M., Bogucki D., Jarosik P., Blachowski B. (2025). Structural Component Identification and Damage Localization of Civil Infrastructure Using Semantic Segmentation. Sensors.

[78] Xu B.R., Shao W.X., Dong X.H. (2025). Drone-Based Wall Crack Detection Using Model-Agnostic Meta-Learning. IEEE Trans. Autom. Sci. Eng..

[79] Yang G.D., Zhao B.Y., Zhang J.H., Wen J.J., Li Q.X., Lei L., Chen X., Chen B.M. (2025). Det-Recon-Reg: An Intelligent Framework Toward Automated UAV-Based Large-Scale Infrastructure Inspection. IEEE Trans. Instrum. Meas..

[80] Zhang L., Gong L.L., Wang L., Wang Z., Yan S. (2025). A Building Crack Detection UAV System Based on Deep Learning and Linear Active Disturbance Rejection Control Algorithm. Electronics.

[81] He Y.B., Yuan Z.R., Xia X.H., Yang B., Wu H.T., Fu W., Yao W.X. (2024). Local-Global Feature Adaptive Fusion Network for Building Crack Detection. Sensors.

[82] Nguyen C.L., Nguyen A., Brown J., Byrne T., Ngo B.T., Luong C.X. (2024). Optimising Concrete Crack Detection: A Study of Transfer Learning with Application on Nvidia Jetson Nano. Sensors.

[83] Sarhadi A., Ravanshadnia M., Monirabbasi A., Ghanbari M. (2024). Using an improved U-Net plus plus with a T-Max-Avg-Pooling layer as a rapid approach for concrete crack detection. Front. Built Environ..

[84] Wang W.J., Su C., Han G.H. (2025). Enhancement of Motion Blurred Crack Images Based on Conditional Generative Adversarial Network. Arab. J. Sci. Eng..

[85] Cai R.Y., Li J.R., Tan Y., Shou W.C., Butera A. (2024). Automated Geometric Quantification of Building Exterior Wall Cracks Based on Computer Vision. J. Perform. Constr. Facil..

[86] Loverdos D., Sarhosis V. (2024). Pixel-level block classification and crack detection from 3D reconstruction models of masonry structures using convolutional neural networks. Eng. Struct..

[87] Meivel S., Devi K.I., Subramanian A.S., Kalaiarasi G. (2025). Remote Sensing Analysis of the LIDAR Drone Mapping System for Detecting Damages to Buildings, Roads, and Bridges Using the Faster CNN Method. J. Indian Soc. Remote Sens..

[88] Tse K.W., Pi R.D., Yang W.Y., Yu X., Wen C.Y. (2024). Advancing UAV-Based Inspection System: The USSA-Net Segmentation Approach to Crack Quantification. IEEE Trans. Instrum. Meas..

[89] Wang J.H., Ueda T., Wang P.J., Li Z.B., Li Y. (2025). Building damage inspection method using UAV-based data acquisition and deep learning-based crack detection. J. Civ. Struct. Health Monit..

[90] Zhong J.W., Fan Y.L., Zhao X.G., Zhou Q., Xu Y. (2024). Multi-Type Structural Damage Image Segmentation via Dual-Stage Optimization-Based Few-Shot Learning. Smart Cities.

[91] Babu B.P., Khandagale S., Shinde V., Gargote S., Bingi K. (2023). Enhancing Infrastructure Safety: A UAV-Based Approach for Crack Detection. Eng. J..

[92] Bhattacharya G., Puhan N.B., Mandal B. (2022). Kernelized dynamic convolution routing in spatial and channel interaction for attentive concrete defect recognition. Signal Process. Image Commun..

[93] Buatik A., Thansirichaisree P., Kalpiyapun P., Khademi N., Pasityothin I., Poovarodom N. (2024). Mosaic crack mapping of footings by convolutional neural networks. Sci. Rep..

[94] Chen K.W., Reichard G., Xu X., Akanmu A. (2021). Automated crack segmentation in close-range building facade inspection images using deep learning techniques. J. Build. Eng..

[95] Choi D., Bell W., Kim D., Kim J. (2021). UAV-Driven Structural Crack Detection and Location Determination Using Convolutional Neural Networks. Sensors.

[96] Dunphy K., Sadhu A., Wang J.F. (2022). Multiclass damage detection in concrete structures using a transfer learning-based generative adversarial networks. Struct. Control Health Monit..

[97] Egodawela S., Gostar A.K., Buddika H., Dammika A.J., Harischandra N., Navaratnam S., Mahmoodian M. (2024). A Deep Learning Approach for Surface Crack Classification and Segmentation in Unmanned Aerial Vehicle Assisted Infrastructure Inspections. Sensors.

[98] Gwon G.H., Lee J.H., Kim I.H., Baek S.C., Jung H.J. (2023). Image-to-Image Translation-Based Structural Damage Data Augmentation for Infrastructure Inspection Using Unmanned Aerial Vehicle. Drones.

[99] Jiang Y.H., Han S.S., Bai Y. (2021). Building and Infrastructure Defect Detection and Visualization Using Drone and Deep Learning Technologies. J. Perform. Constr. Facil..

[100] Jin T., Zhang W., Chen C.L., Chen B., Zhuang Y.Z., Zhang H. (2023). Deep-Learning- and Unmanned Aerial Vehicle-Based Structural Crack Detection in Concrete. Buildings.

[101] Kim B., Natarajan Y., Preethaa K.R.S., Song S., An J., Mohan S. (2024). Real-time assessment of surface cracks in concrete structures using integrated deep neural networks with autonomous unmanned aerial vehicle. Eng. Appl. Artif. Intell..

[102] Kumar P., Batchu S., Swamy S.N., Kota S.R. (2021). Real-Time Concrete Damage Detection Using Deep Learning for High Rise Structures. IEEE Access.

[103] Kung R.Y., Pan N.H., Wang C.C.N., Lee P.C. (2021). Application of Deep Learning and Unmanned Aerial Vehicle on Building Maintenance. Adv. Civ. Eng..

[104] Le T.T., Nguyen V.H., Le M.V. (2021). Development of Deep Learning Model for the Recognition of Cracks on Concrete Surfaces. Appl. Comput. Intell. Soft Comput..

[105] Lee K., Lee S., Kim H. (2022). Bounding-box object augmentation with random transformations for automated defect detection in residential building facades. Autom. Constr..

[106] Lee T.H., Kim J.H., Lee S.J., Ryu S.K., Joo B.C. (2023). Improvement of Concrete Crack Segmentation Performance Using Stacking Ensemble Learning. Appl. Sci..

[107] Wang J.H., Wang P.J., Qu L., Pei Z., Ueda T. (2024). Automatic detection of building surface cracks using UAV and deep learning-combined approach. Struct. Concr..

[108] Xiang C., Wang W., Deng L., Shi P., Kong X. (2022). Crack detection algorithm for concrete structures based on super-resolution reconstruction and segmentation network. Autom. Constr..

[109] Yao Z.L., Jiang S., Wang S., Wang J.J., Liu H., Narazaki Y., Cui J., Spencer B.F. (2024). Intelligent crack identification method for high-rise buildings aided by synthetic environments. Struct. Des. Tall Spec. Build..

[110] Yoon J., Shin H., Kim K., Lee S. (2024). CNN- and UAV-Based Automatic 3D Modeling Methods for Building Exterior Inspection. Buildings.

[111] Zhang X.H., Huang H.F. (2024). LCSNet: Light-Weighted Convolution-Based Segmentation Method with Separable Multi-Directional Convolution Module for Concrete Crack Segmentation in Drones. Electronics.

[112] Li F., Qian H., Xiong J.C., Chen W.Y., Umar M. (2025). Optimizing Concrete Defect Classification Model with a Novel Comprehensive Dataset. Struct. Control Health Monit..

[113] Li Q.X., Peng X., Zhong X.G., Xiao X.Y., Wang H., Zhao C., Zhou K. (2024). Quantitative identification of debonding defects in building façades based on UAV-thermography using a two-stage network integrating dual attention mechanism. Infrared Phys. Technol..

[114] Tan J.L., Li Y.L. (2025). Damage Identification of Industrial Building Grid Structures Based on Perceptual Fuzzy Systems. IEEE Access.

[115] Santos R., Ribeiro D., Lopes P., Cabral R., Calcada R. (2022). Detection of exposed steel rebars based on deep-learning techniques and unmanned aerial vehicles. Autom. Constr..

[116] Yang K., Tan M., Liao H.T., Azucena J.C.H., Yuan J.L., Ham R. (2025). An Attention-Enhanced YOLOv8 Architecture for Leakage Detection in Building Systems. J. Perform. Constr. Facil..

[117] Zhang J.Y., Huang L., Guan Y.J. (2025). Real-time defect detection in concrete structures using attention-based deep learning and GPR imaging. Sci. Rep..

[118] Dabetwar S., Padhye R., Kulkarni N.N., Niezrecki C., Sabato A. (2023). Performance evaluation of deep learning algorithms for heat loss damage classification in buildings from UAV-borne infrared images. J. Build. Eng..

[119] Kang S., Jiao M.H., Wan H.T., Yu Y., Yin J.H., Zhao T.J. (2025). Building damage assessment based on an enhanced PSPNet network and UAV images. Constr. Build. Mater..

[120] Wang Y.L., Feng W.Q., Jiang K., Li Q.C., Lv R.P., Tu J.H. (2023). Real-Time Damaged Building Region Detection Based on Improved YOLOv5s and Embedded System From UAV Images. IEEE J. Sel. Top. Appl. Earth Obs. Remote Sens..

[121] Geetha G.K., Yang H.J., Sim S.H. (2023). Fast Detection of Missing Thin Propagating Cracks during Deep-Learning-Based Concrete Crack/Non-Crack Classification. Sensors.

[122] Agyemang I.O., Zeng L.Y., Chen J.W., Adjei-Mensah I., Acheampong D. (2024). Multi-visual modality micro drone-based structural damage detection. Eng. Appl. Artif. Intell..

[123] Agyemang I.O., Zhang X.L., Acheampong D., Adjei-Mensah I., Kusi G.A., Mawuli B.C., Agbley B.L.Y. (2022). Autonomous health assessment of civil infrastructure using deep learning and smart devices. Autom. Constr..

[124] Yao G., Sun W.T., Yang Y., Sun Y.J., Xu L.J., Zhou J. (2022). Chromatic Aberration Identification of Fair-Faced Concrete Research Based on Multi-Scale Lightweight Structured Data Algorithm. Front. Mater..

[125] Yigit A.Y., Uysal M. (2024). Automatic crack detection and structural inspection of cultural heritage buildings using UAV photogrammetry and digital twin technology. J. Build. Eng..

[126] Jiang Y.H., Han S.S., Bai Y. (2022). Scan4Facade: Automated As-Is Facade Modeling of Historic High-Rise Buildings Using Drones and AI. J. Archit. Eng..

[127] Loverdos D., Sarhosis V. (2023). Image2DEM: A geometrical digital twin generator for the detailed structural analysis of existing masonry infrastructure stock. SoftwareX.

[128] Levine N.M., Narazaki Y., Spencer B.F. (2023). Development of a building information model-guided post-earthquake building inspection framework using 3D synthetic environments. Earthq. Eng. Eng. Vib..

[129] Levine N.M., Spencer B.F. (2022). Post-Earthquake Building Evaluation Using UAVs: A BIM-Based Digital Twin Framework. Sensors.

[130] Shen W.J., Wang C., Zhu C., Wang D.F., Chen Z.B. (2026). Reverse digital twin mapping of building envelope anomalies based on UAV infrared point clouds. Measurement.

[131] Polania D.R., Tondolo F., Osello A., Piras M., Di Pietra V., Grasso N. (2025). Bridges monitoring and assessment using an integrated bim methodology. Innov. Infrastruct. Solut..

[132] Hao S.J., Huang X.H., Duan Z., Hou J., Chen W., Cai L.X. (2025). Research on the Method for Pairing Drone Images with BIM Models Based on Revit. Drones.

[133] Tan Y., Li G., Cai R.Y., Ma J., Wang M.Z. (2022). Mapping and modelling defect data from UAV captured images to BIM for building external wall inspection. Autom. Constr..

[134] Zhang J.H., Zhao B.Y., Yang G.D., Zhou X.K., Huang Y.J., Gao C.X., Chen X., Chen B.M. (2025). AI-empowered digital twin modeling for high-precision building defect management integrating UAV and GeoBIM. Build. Simul..

[135] Yang L., Liu K.J., Ou R.S., Qian P., Wu Y.J., Tian Z., Zhu C.P., Feng S.N., Yang F. (2024). Surface Defect-Extended BIM Generation Leveraging UAV Images and Deep Learning. Sensors.

[136] Nieto-Julián E., Robador M.D., Moyano J., Bruno S. (2025). Semantic HBIM for Heritage Conservation: A Methodology for Mapping Deterioration and Structural Deformation in Historic Envelopes. Buildings.

[137] Hamdan A., Taraben J., Helmrich M., Mansperger T., Morgenthal G., Scherer R.J. (2021). A semantic modeling approach for the automated detection and interpretation of structural damage. Autom. Constr..

[138] Han C.L. (2025). BIM-Integrated UAV-Based Defect Detection with Edge Computing for Infrastructure Inspection. Internet Technol. Lett..

[139] Jang K., Kim J.W., Ju K.B., An Y.K. (2021). Infrastructure BIM Platform for Lifecycle Management. Appl. Sci..

[140] Ragusa E., Taccioli T., Canepa A., Zunino R., Gastaldo P. (2024). Design and Implementation of Tiny Deep Neural Networks for Landing Pad Detection on UAVs. IEEE Access.

[141] Zhu G.J., Shen S.L., Yao J.J., Wang M.H., Zhuang J.F., Fan Z. (2025). Automatic lightweight networks for real-time road crack detection with DPSO. Adv. Eng. Inform..

[142] Gao P.P., Wu T.T., Song C.H. (2024). Cloud-Edge Collaborative Strategy for Insulator Recognition and Defect Detection Model Using Drone-Captured Images. Drones.

